# The plasticity and metabolic reprogramming of macrophage in ulcerative colitis

**DOI:** 10.3389/fimmu.2026.1903462

**Published:** 2026-09-04

**Authors:** Yi Zhang, Dingquan Yang, Junnan Jiang, Dongxu Wang, Fujian Ji, Ziqian Sun

**Affiliations:** 1 Department of Gastrointestinal Colorectal Surgery, China-Japan Union Hospital of Jilin University, Jilin University, Changchun, Jilin, China; 2 Department of Laboratory Animals, College of Animal Sciences, Jilin University, Changchun, Jilin, China; 3 Obstetrics and Gynecology Department, The Second Hospital of Jilin University, Changchun, Jilin, China

**Keywords:** inflammation, macrophage polarization, metabolic reprogramming, mitochondrial dynamics, ulcerative colitis

## Abstract

Macrophages exhibit phenotypic plasticity, which plays a role in driving tissue injury and promoting mucosal repair in ulcerative colitis (UC). Intestinal macrophages originate from both embryo-derived resident cells and circulating monocytes. Macrophage polarization influences inflammatory responses and is regulated by cytokines, chemokines, growth factors, and adhesion molecules. Tissue-specific metabolic reprogramming governs macrophage plasticity. Glucose metabolic reprogramming drives M1-like polarization. During glycolysis, mitochondria influence reactive oxygen species (ROS) production which alters the fate of macrophages in UC. Moreover, the communication of cell to cell can also regulate macrophage plasticity. Transcriptomics, proteomics, and single-cell sequencing can be employed to dissect the phenotypes and heterogeneity of macrophage polarization. Numerous clinical trials suggest that regulating macrophage polarization, inhibiting excessive activation of macrophages, blocking the abnormal recruitment of macrophages to intestinal inflammatory sites, macrophage-directed cell therapy, and combination therapy can effectively treat patients with UC. Indeed, the macrophage polarization is characterized by ROS, microbial community and bacterial products in inflammation of the colonic mucosa which is regulated by metabolic programming of macrophages. Indeed, macrophage polarization is characterized by ROS, microbial community, and bacterial products in the inflamed colonic mucosa. The macrophage polarization process is tightly regulated by the metabolic programming. In summary, this review analyzes macrophage plasticity and functional diversity, as well as the metabolic reprogramming and microenvironmental signals that shape macrophage polarization, and offers new insights for macrophage-based clinical interventions in UC.

## 1 Introduction

Currently, UC is recognized as a major form of inflammatory bowel disease (IBD) and significantly impairs patients’ quality of life (1). It is characterized by chronic idiopathic inflammation that continuously affects the colonic mucosa (2). The intestinal tissue damage observed in UC is primarily driven by a dysregulated immune response (3). During this response, a variety of immune cells are involved in the pathogenesis of UC, including neutrophils, dendritic cells, monocytes, natural killer (NK) cells, and macrophages. Indeed, the macrophages and other innate immune cells can release pro-inflammatory cytokines, such as tumor necrosis factor-α (TNF-α) and interleukin-1β (IL-1β), and ROS which influence the inflammatory response (4, 5).

As key components of the innate immune system, macrophages play a central role in the pathogenesis of IBD, particularly UC (6). Macrophages were first identified in the 19th century, a discovery that laid the foundation for the study of phagocytosis and contributed to the development of cellular immunology (7, 8). The intestinal macrophages are derived from embryo-derived resident cells and circulating monocytes. Circulating monocytes migrate into various tissues, such as the intestine, where they differentiate into macrophages (9, 10). Macrophage plasticity maintains intestinal homeostasis and participates in inflammatory responses and tissue repair processes in the gut (4). Metabolic reprogramming, particularly enhanced glycolysis, contributes to macrophage polarization (11).

Macrophages within the intestinal tissue exhibit considerable heterogeneity. Previous studies have primarily focused on epithelium-associated and lamina propria-associated macrophages (LPAMs) in both the large and small intestines (10, 12). LPAMs are initially derived from fetal precursors. However, they are rapidly replaced by short-lived, monocyte-derived macrophages in a process dependent on C-C chemokine receptor 2 (CCR2) and supported by an active microbiota (12, 13). These LPAMs are essential for maintaining intestinal barrier homeostasis. Tissue-resident macrophages help regulate the differentiation of incoming monocytes by expressing immunomodulatory cytokines such as IL-10 and transforming growth factor-beta (TGF-β) (14).

In this review, macrophage plasticity is analyzed. Furthermore, the metabolic reprogramming, cytokines, and microenvironmental signals involved in macrophage polarization are investigated. Moreover, the application of omics in macrophages is discussed. Finally, the potential strategies that may be adopted for modulating macrophage function in clinical practice are highlighted.

## 2 Ontogeny and fate of macrophages in colitis

Macrophages play a central role in maintaining systemic homeostasis and regulating immune processes in UC. Moreover, as tissue-resident macrophages across organ systems, macrophages differentiate into distinct functional subtypes, broadly categorized as classically activated (M1-like) and alternatively activated (M2-like) macrophages under varying microenvironmental signals (15, 16).

### 2.1 Divergent differentiation fates of monocytes

Indeed, the intestinal macrophages are primarily derived from embryo-derived resident cells and circulating monocytes (17). Embryo-derived resident macrophages can maintain tissue homeostasis. The circulating monocytes, Ly6C hi monocytes, have the ability to continuously enter the intestinal tissue and differentiate into mature resident macrophages with anti-inflammatory and immune-regulatory functions (18). However, monocytes do not differentiate into mature resident macrophages under inflammatory conditions, instead become arrested in pro-inflammatory state. Previous study has shown that ROS are abnormally elevated and create an oxidative stress microenvironment in the UC lesions. Superoxide dismutase 2 (SOD2), which is an ROS-scavenging enzyme, is scarcely expressed in mature tissue-resident macrophages (19). In contrast, recently recruited inflammatory macrophages normally express SOD2. Thus, the mature tissue-resident macrophages which lack expression of SOD2 suffer extensive depletion in the UC lesions. Interestingly, the remaining mature tissue-resident macrophages, which have altered gene expression, have a function that promote tissue repair (20). Compared with Crohn’s disease (CD), the ROS-driven depletion of tissue-resident macrophages has been specifically demonstrated in UC lesions (19, 21). These macrophage populations may experience different selective pressures imposed by the chronic transmural inflammation-driven fibrotic microenvironment in CD.

### 2.2 M1-like and M2-like macrophages in UC

The intestinal mucosal barrier is essential for colorectal homeostasis (22). Disruption of the gut microbiota, invasion of pathogens, or immune dysregulation can compromise this barrier, prompting macrophages to transition from the M0 state and polarize into the pro-inflammatory M1-like phenotype. This process is induced by stimuli such as lipopolysaccharide (LPS), interferon-γ (IFN-γ), and TNF-α (23). M1-like macrophages secrete a large number of inflammatory factors, such as IL-1β (24), interleukin-6 (IL-6) (25), interleukin-12 (IL-12) (26), TNF-α (27), nitric oxide (NO) (28), and reactive oxygen species (ROS) (29), to exert pro-inflammatory effects. M1-like macrophages express the cell surface markers CD40, CD80, CD86, and major histocompatibility complex class II receptor (MHC-IIR) (30). Upon activation, M1-like macrophages may contribute to tissue damage by excessively phagocytosing intestinal epithelial cells or degrading the extracellular matrix through the release of matrix metalloproteinases (MMPs, such as MMP-9) (31). These lead to mucosal erosion and ulcer formation.

Upon inflammation, immune cells secrete cytokines that can promote macrophage polarization toward the M2-like phenotype (32). Moreover, Th2 cells and innate lymphoid cells type 2 (ILC2s) secrete IL-4 and IL-13, while regulatory T cells (Tregs) and macrophages themselves secrete IL-10. These M2-like macrophages express surface markers, including CD163, CD204, and CD206. Furthermore, they release anti-inflammatory factors, such as IL-10 and TGF-β, following the onset of intestinal inflammation. Their primary functions include suppressing excessive inflammatory responses and promoting mucosal repair. Thus, M2-like macrophages contribute to the restoration of intestinal homeostasis in UC (33). In addition, M2-like macrophages also facilitate the clearance of apoptotic cells through efferocytosis, which creates a microenvironment conducive to tissue regeneration (34).

The M2-like macrophage subtypes, M2a, M2b, M2c, and M2d, can be polarized by distinct cytokines and signaling pathways (35). These M2-like macrophages exert anti-inflammatory functions, limit mucosal damage, promote mucosal repair, and maintain vascular homeostasis (**Table 1**). However, these M2-like subtypes were defined by specific cytokine combinations, signaling pathways and transcription factors *in vitro*. Thus, the human UC tissue microenvironment remains to be fully established to confirm the functional relevance of these M2-like subtypes.

**Table 1:** The origin, differentiation and functional characteristics of macrophages in ulcerative colitis.

Feature classification	Steady-state resident macrophages (resident, embryonic-derived)	M1 macrophages (classically activated)	M2 macrophage subtypes			
			M2a (Th2/repair)	M2b (immunoregulatory)	M2c (deactivated / desensitized)	M2d
Origin/Precursor	Yolk sac / fetal liver red pulp progenitors; seed the gut at birth, locally self−maintained (independent of bone marrow)					
Differentiation/Polarizing Stimuli	Local IL.34, TGF.β, microbial products maintain the phenotype	IFN.γ, LPS; GM.CSF	IL.4, IL.13	Immune complexes (IC), LPS, IL.1β	IL.10, TGF.β, apoptotic cells, glucocorticoids	IL-6 and IL-10
Surface Markers	Mouse: CX3CR1^high^, CD64^+^, MHC.II^+^, F4/80^int^, Tim4^+^ Human: CD163^+^, CD206^low^, CX3CR1^high^, HLA.DR^+^	Mouse: CD86^high^, CD80^high^, MHC-II^high^, CD16/32^+^ Human: CD80^+^, CD86^+^, HLA.DR^high^	Mouse: CD206^+^, Arg1^+^, CD301 (Mgl1) Human: CD206^+^, CD209^+^, IL.1RII^+^	Mouse: CD86^+^, CD206^low^, TLR1/8^+^ Human: CD163^+^, CD86^+^, HLA.DR^+^	Mouse: CD163^+^, CD206^+^, MerTK^+^ Human: CD163^high^, CD206^high^, MerTK^+^, TLR2/4^low^	Mouse/human shared: CD206^+^, CD163^+^, PD.L1^+^, CD209^+^ (human), MerTK^+^; CD274 and VEGFR1
Secreted Factors	IL.10, PGE2, BMP2, TGF.β; IL.1β/TNF	TNF, IL.1β, IL.6, IL.12, IL.23, CXCL9/10/11, iNOS, ROS	IL.10, TGF.β, CCL17, CCL22, CCL24, PDGF, VEGF	IL.1β, IL.6, IL.10, TNF, CCL1	IL.10, TGF.β, PGE2, MerTK, CCL18	IL−10, TGF−β, VEGF, MMP−2/9, CXCL1/8, IL−6, Arginase−1, IL−12
Major Functions	Immunosuppression: promote Treg, suppress Th1/Th17. Barrier maintenance: clear apoptotic cells and escaped bacteria (silent phagocytosis). Tissue repair: support epithelial stem cells, regulate motility.	Intracellular killing (Listeria, Mycobacteria). Anti.tumor (direct cytotoxicity, promote Th1). Over.activation leads to chronic inflammation (e.g., active Crohn’s disease).	Anti.parasite (helminths). Pro.fibrotic (collagen deposition). Angiogenesis (wound healing). Suppress Th1 responses.	Mixed regulation: both pro. and anti.inflammatory. Th2 bias (via CCL1). May be involved in allergy and autoimmunity regulation.	Potent anti.inflammation: efferocytosis. Tissue remodeling (MMP inhibition). Immune tolerance (induce Treg, suppress DC maturation). Pro.tumor (main TAM phenotype).	suppress anti.tumor immunity (via PD.L1/IL.10/TGF.β), promote angiogenesis (VEGF), tumor invasion and metastasis (MMPs). Chemoresistance (via IL.6 etc.)
Metabolic Features	OXPHOS dominant, low glycolysis; FAO dependent, moderate mitochondrial activity; no succinate accumulation	Glycolysis.dominant (Warburg): high glucose uptake, lactate accumulation; PPP for NADPH; TCA break → succinate accumulation stabilizes HIF.1α; enhanced fatty acid synthesis	OXPHOS + FAO: intact mitochondrial respiration; enhanced glutaminolysis → UDP.GlcNAc for CD206 glycosylation; arginine → polyamines/proline	Mixed glycolysis + OXPHOS; glutamine dependent; low FAO	OXPHOS highly FAO dependent; high MerTK promotes lipid phagocytosis; increased mitochondrial cristae density	Mixed metabolism: partly OXPHOS and FAO (similar to M2c), but can upregulate aerobic glycolysis (HIF.1α.driven) in the TME, along with enhanced arginine and glutamine metabolism
Special Role in the Gut	• Constitute >90% under homeostasis • Highly dependent on IL.10 signaling (IL.10R mutation → early.onset colitis) • Do not migrate to lymph nodes	• Dominant in active IBD, produce large amounts of TNF (target of anti.TNF therapy) • Positive feedback with Th1/Th17 cells	• Transiently appear in recovery phase of IBD • Promote fibrostenosis in chronic phase (Crohn’s disease) • IL.4 sources are rare in the gut; IL.13 is more common	• Less studied in the gut • May be seen in monocytes after LPS tolerance • Associated with “atypical” inflammatory responses in some IBD patients	• Dominant in remission phase of IBD • CD163^+^ cells increased in UC but functionally impaired • Main phenotype of tumor.associated macrophages (TAM)	• Abundant in colitis−associated cancer (CAC) models and human colorectal cancer • Correlated with poor prognosis, metastasis, and immune therapy resistance • Gut microbial metabolites (e.g., secondary bile acids) can promote M2d polarization • Targeting CSF−1R or CCR2 reduces M2d/TAM

UC, Ulcerative Colitis; M1, M1 Macrophages; M2a / M2b / M2c / M2d:M2a / M2b / M2c / M2d Macrophages; IL-34, Interleukin-34; TGF-β, Transforming Growth Factor Beta; IFN-γ, Interferon Gamma; LPS, Lipopolysaccharide; GM-CSF, Granulocyte-Macrophage Colony-Stimulating Factor; IL-4, Interleukin-4; IL-13, Interleukin-13; IC, Immune Complexes; IL-1β, Interleukin-1 Beta; IL-10, Interleukin-10; IL-6, Interleukin-6; CX3CR1, C-X3-C Motif Chemokine Receptor 1; CD64, Cluster of Differentiation 64; MHC-II, Major Histocompatibility Complex Class II; F4/80, F4/80 Antigen (*mouse macrophage marker; also associated with ADGRE1/EMR1*); Tim4, T-cell Immunoglobulin and Mucin Domain-containing Protein 4; HLA-DR, Human Leukocyte Antigen - DR isotype; CD86, Cluster of Differentiation 86; CD80, Cluster of Differentiation 80; CD16/32, Cluster of Differentiation 16/32 (commonly referring to FcγRIII/FcγRII in mice); Arg1, Arginase 1; CD301, Cluster of Differentiation 301; Mgl1, Macrophage Galactose-type Lectin 1; CD209, Cluster of Differentiation 209; IL-1RII, Interleukin-1 Receptor Type II; TLR1/8, Toll-like Receptor 1/8; MerTK, MER Tyrosine Kinase; TLR2/4, Toll-like Receptor 2/4; PD-L1, Programmed Death-Ligand 1; CD274, Cluster of Differentiation 274; VEGFR1, Vascular Endothelial Growth Factor Receptor 1; PGE2, Prostaglandin E2; BMP2, Bone Morphogenetic Protein 2; TNF, Tumor Necrosis Factor; IL-12, Interleukin-12; IL-23, Interleukin-23; CXCL9, C-X-C Motif Chemokine Ligand 9; CXCL10, C-X-C Motif Chemokine Ligand 10; CXCL11, C-X-C Motif Chemokine Ligand 11; iNOS, Inducible Nitric Oxide Synthase; ROS:Reactive Oxygen Species; CCL17:C-C Motif Chemokine Ligand 17; CCL22:C-C Motif Chemokine Ligand 22; CCL24:C-C Motif Chemokine Ligand 24; PDGF:Platelet-Derived Growth Factor; VEGF:Vascular Endothelial Growth Factor; CCL1, C-C Motif Chemokine Ligand 1; CCL18:C-C Motif Chemokine Ligand 18; Treg, Regulatory T Cells; Th1, T Helper 1 Cells; Th2, T Helper 2 Cells; Th17, T Helper 17 Cells; DC, Dendritic Cells; TAM, Tumor-Associated Macrophages; MMP, Matrix Metalloproteinase; MMP-2, Matrix Metalloproteinase-2; MMP-9, Matrix Metalloproteinase-9; OXPHOS, Oxidative Phosphorylation; FAO, Fatty Acid Oxidation; PPP, Pentose Phosphate Pathway; NADPH, Nicotinamide Adenine Dinucleotide Phosphate (Reduced Form); TCA, Tricarboxylic Acid Cycle; HIF-1α, Hypoxia-Inducible Factor 1-alpha; UDP-GlcNAc, Uridine Diphosphate N-Acetylglucosamine; IL-10R, Interleukin-10 Receptor;IBD, Inflammatory Bowel Disease; CAC, Colitis-Associated Cancer; TME, Tumor Microenvironment; CSF-1R, Colony-Stimulating Factor 1 Receptor; CCR2, C-C Motif Chemokine Receptor 2.

### 2.3 The heterogeneity of macrophage in the UC intestine

Recently, the classical M1/M2 polarization framework has been challenged by single-cell transcriptomic analyses that reveal extensive macrophage heterogeneity in the inflamed colon of UC patients (21, 36, 37). Tissue-resident macrophages have been identified as embryo-derived or long-lived monocyte-derived cells that express high levels of CD163, C1QA, SELENOP, and LYVE1 (13, 38). These tissue-resident macrophages maintain tissue integrity, clear apoptotic cells *via* efferocytosis, and support epithelial stem cell renewal under homeostatic conditions (39, 40). Due to oxidative stress-driven depletion, their numbers are dramatically reduced in active lesions in UC. Moreover, the inflammatory monocyte-derived macrophages arise from newly recruited Ly6C hi monocytes and are the dominant macrophage population in active UC lesions. These macrophages express *FCGR3A* (CD16), *TREM1*, *IL1B*, and *TNF*, produce amounts of IL-1β, TNF-α, and ROS, acting as key drivers of inflammation-mediated tissue injury (11, 21). In addition, disease-associated macrophages (DAMs), as a subset of macrophages in chronically inflamed tissues, and are thought to represent an adaptation to sustained inflammatory and tissue-damaging signals (36). The Single-cell RNA sequencing (scRNA-seq) study has also identified the transitional and reparative populations of macrophage subsets. These cells, including *IL1RN*^+^ macrophages, express high levels of the IL-1 receptor antagonist, and *Cadm1*^+^ macrophages that have been linked to mucosal healing during the resolution phase (41). Rather than simply aiming to block M1-like or induce M2-like polarization, therapeutic strategies should consider this heterogeneity and aim to selectively deplete pathogenic inflammatory macrophages, preserve or restore tissue-resident macrophages, and promote the emergence of reparative transitional subsets in UC.

Considering that macrophage polarization derives using murine bone marrow-derived macrophages stimulated with LPS and IFN-γ (for M1-like) or IL-4/IL-13 (for M2-like), may not fully recapitulate the complexity of human UC. The macrophages are exposed to a complex mixture of microbial products, host-derived signals, and metabolic stresses simultaneously in human UC. Furthermore, significant species-specific differences exist between mouse and human macrophages in the expression of polarization markers and metabolic programming. Thus, this review focuses on human UC, examining how these intestine-specific factors shape macrophage plasticity, particularly microbiota-derived metabolites and the hypoxic niche.

## 3 Regulatory factors in macrophage polarization of UC

Macrophages display remarkable phenotypic plasticity through their influence on inflammation and tissue repair in UC (42). This functional adaptability extends beyond the initial differentiation of monocytes into M1-like or M2-like activated macrophages, encompassing potential reprogramming of established phenotypes.

### 3.1 The cytokines in macrophage polarization

#### 3.1.1 M1-like polarizing cytokines

The pro-inflammatory factors, IFN-γ, TNF-α, IL-1β, and IL-12, promote polarization toward M1-like macrophages in UC. IFN-γ which is derived from activated Th1, NK, and natural killer T (NKT) cells, can activate the signal transducer and activator of transcription 1 (STAT1) pathway (17). IFN-γ upregulates M1 markers, such as iNOS and CD86, while suppressing the M2-like pathway, such as IL-4/STAT6 (43). TNF-α which is secreted by macrophages, Th1 cells, and intestinal epithelial cells, can enhance M1 polarization *via* nuclear factor-kappa B (NF-κB) and mitogen-activated protein kinase (MAPK) signaling. IL-1β, which is derived from activated macrophages, monocytes, and epithelial cells, has a role in activating NF-κB, and promotes adhesion molecule expression to recruit neutrophils and additional monocytes to sites of inflammation. IL-12, which is mainly derived from dendritic cells and activated macrophages, can drive M1 polarization by activating STAT4 in UC (5).

#### 3.1.2 M2-like polarizing cytokines

The anti-inflammatory cytokines, IL-4, IL-13, IL-10, and TGF-β, drive polarization of macrophages toward an M2-like phenotype. These cytokines inhibit inflammation, and promote tissue repair (44). IL-4 activates STAT6, upregulating M2-like markers, while inhibiting the NF-κB-mediated M1-like pathway. IL-4 promotes epithelial proliferation and mucosal repair (45). IL-13 reduces the infiltration of inflammatory cells, including neutrophils and macrophages, and restores mucosal architecture through STAT1/STAT6 signaling (46). IL-10, through the activation of STAT3, directly suppresses the transcription of pro-inflammatory cytokines (47). Simultaneously, it maintains intestinal homeostasis by reinforcing the M2-like polarization program, which collectively contributes to the resolution of inflammation and tissue repair. TGF-β promotes M2-like polarization *via* the Smad2/3 pathway (48).

### 3.2 Chemokines in macrophage recruitment

Chemokines derived from epithelial cells, fibroblasts, and activated immune cells (such as T cells and macrophages) orchestrate macrophage and monocyte recruitment to inflamed intestinal sites in UC. CCL2/MCP-1 is the principal chemokine that recruits circulating monocytes for differentiation into intestinal macrophages. Overexpression of CCL2/MCP-1 leads to macrophage accumulation in the deep intestinal wall, exacerbating tissue damage and fibrosis (17). CCL3/MIP-1α synergizes with CCL2 to enhance recruitment and activate pro-inflammatory functions. While CCL4/MIP-1β maintains mucosal macrophage colonization and CCL5/RANTES activates CCR5 on macrophages to promote aggregation and the release of pro-inflammatory factors, such as TNF-α and IL-1β. CXCL8/IL-8 recruits neutrophils and subsequently induces monocyte migration *via* the CXCL8-C-X-C motif chemokine receptor (CXCR) 1/2 axis. CXCL12/SDF-1α mobilizes monocytes from bone marrow to blood and intestine. These monocytes differentiate into macrophages that exhibit repair functions through CXCR4/CXCR7 (49, 50). CX3CL1/Fractalkine adheres to circulating monocytes to facilitate their transendothelial migration into the mucosa. While CCL7 assists CCL2 in initiating mucosal inflammation (51). Collectively, CCL2 acts as the primary recruiter, CCL3/4/5 serve as co-factors, and CXCL8/CXCL12 play auxiliary roles in acute or recovery phases, coordinately regulating macrophage recruitment, activation, and function in UC.

### 3.3 Growth factors in macrophage polarization

Colony-Stimulating Factors (CSFs), vascular endothelial growth factor (VEGF), and epidermal growth factor (EGF) regulate the survival, proliferation, activation, and functional differentiation of macrophages in UC. CSFs are key growth factors that regulate the lineage development, proliferation, and function of macrophages. Moreover, granulocyte-macrophage colony-stimulating factor (GM-CSF) promotes bone marrow-derived monocytes to migrate to intestinal inflammatory sites and further differentiate into mature macrophages (52). Colony-stimulating factor 1 (CSF1) regulates the differentiation of monocytes into tissue-resident macrophages and maintains homeostasis of macrophages (53). VEGF, such as VEGFA, regulates macrophage functions through paracrine action and promotes vascular repair. A study has demonstrated that VEGFA can enhance the ability of macrophage to influence intestinal inflammation in UC (54). In addition, EGF regulates the proliferation of intestinal epithelial cells to participate in repair. A report suggested that EGF upregulates the expression of phagocytosis-related proteins and accelerates the clearance of necrotic cells (55).

### 3.4 Adhesion molecules in macrophage communication

The integrin family, selectin family, immunoglobulin family are the adhesion molecules that mediate cell-to-cell communication. Integrins, such as α4β7, have a role in blocking recruitment of T-cells and influencing the migration of pro-inflammatory monocytes to the intestine in UC (56). Previous studies have demonstrated that selectins mediate leukocyte rolling, adhesion, and subsequent transmigration across the endothelium during inflammation. In particular, E-selectin (CD62E) and P-selectin (CD62P) expressed on activated endothelial cells contribute to the recruitment of inflammatory cells, including neutrophils and macrophages, to sites of inflammation (57, 58). Adhesion molecules of the immunoglobulin superfamily mediate direct cell-to-cell contact through immunoglobulin-like domains. Beyond their adhesive functions, several members of this superfamily, including ICAM-1, VCAM-1, and PECAM-1, can transduce intracellular signals, thereby initiating downstream signaling cascades upon ligand engagement (59). Thus, immunoglobulin superfamily members not only mediate cell-cell contact but also transduce signals that directly influence macrophage polarization.

**Figure 1:**
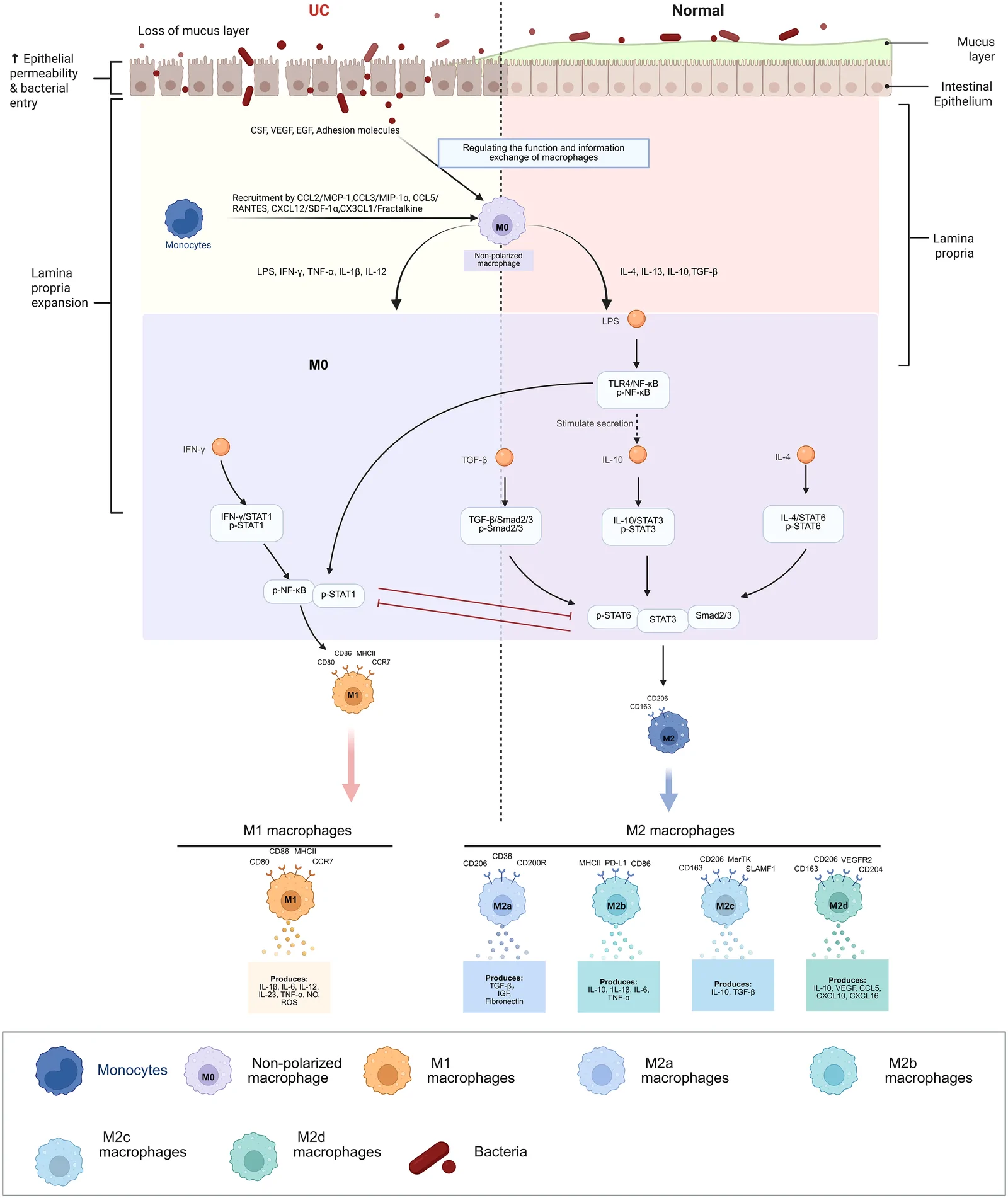
Macrophage polarization and its regulatory mechanisms in UC. Pro-inflammatory factors, including interferon-gamma (IFN-γ) and tumor necrosis factor-alpha (TNF-α), drive M1-like macrophage polarization via signaling pathways including NF-κB, MAPK, and STAT1, thereby exerting pro-inflammatory effects. Anti-inflammatory factors, including interleukin-4 (IL-4), IL-13, IL-10, and transforming growth factor-beta (TGF-β), promote M2-like macrophage polarization primarily through the signal transducer and activator of transcription 1 (STAT1), STAT6, and STAT3 pathways, functioning to counteract inflammation and promote restoration of the intestinal mucosal architecture. Moreover, M2-like macrophage has four subtypes, M2a, M2b, M2c, and M2d.

These findings indicate that a complex regulatory network with cytokines, chemokines, growth factors, and adhesion molecules coordinates macrophage polarization, recruitment, and function in UC (**Figure 1**).

### 3.5 Macrophage crosstalk with the intestinal immune network

As a central hub, macrophages integrates signals from multiple immune and non-immune cell types within the intestinal microenvironment. Dendritic cells (DCs) regulate macrophage polarization by cell to cell contact. Previous study indicate that Toll-like receptor 4 (TLR4)-activated DCs upregulate CD80/CD86, which engage CD28/CTLA-4 on T cells and indirectly shape the cytokine milieu that influences macrophage polarization (60, 61). Moreover, activated T cells interact with macrophages through CD40-CD40L binding which involves NF-κB signaling. Conversely, regulatory T cells secrete IL-10 and TGF-β, promoting M2-like polarization in chronic inflammation (62, 63). In addition, macrophages recruit neutrophils to inflammatory foci by secreting C-X-C motif chemokine ligand 8 (CXCL8), CXCL1, and CXCL2. Then, these neutrophils release neutrophil extracellular traps (NETs) and ROS. Macrophages subsequently clear apoptotic neutrophils through efferocytosis (64, 65). The damaged epithelial cell-derived damage-associated molecular patterns (DAMPs) activate macrophages, while macrophage-derived factors such as IL-10 and TGF-β promote epithelial proliferation and barrier repair. These epithelial cells are essential for the survival and maintenance of tissue-resident macrophages (17, 45, 66). The complement cascade intersects with macrophage function. Complement components C3a and C5a act as potent chemoattractants and activators of macrophages, while opsonization of pathogens and cellular debris by C3b facilitates macrophage-mediated clearance (67, 68). The intracellular complement activation can influence macrophage polarization state and metabolic reprogramming (69, 70). Thus, these interactions position macrophages as integrators of diverse signals from the broader immune network in UC.

## 4 Signaling pathways and transcription factors in macrophage polarization

### 4.1 Tissue-specific metabolic programming of macrophages in UC

Tissue-specific metabolic programming of macrophages is the core mechanism driving their functional shift from a pro-inflammatory to a reparative state in UC. Glucose metabolic reprogramming is a hallmark feature of pro-inflammatory M1-like macrophages. Initially, for efficient energy production, the macrophages rely on oxidative phosphorylation (OXPHOS) (11). Subsequently, aerobic glycolysis is adopted in the inflammatory and hypoxic microenvironment of UC. In this metabolic reprogramming process of UC, hypoxia-inducible factor 1α (HIF-1α), a key regulatory factor of glycolysis, promotes the expression of glycolytic enzymes, with pyruvate kinase M2 (PKM2) playing a central role (41). In contrast to glucose metabolism, lipid metabolic reprogramming primarily involves fatty acid oxidation (FAO). The lipid metabolic reprogramming supports the anti-inflammatory and reparative functions of M2-like macrophages. For example, the high-mobility group box 1 (HMGB1) protein blocks FAO by inhibiting the expression of carnitine palmitoyltransferase 1A (CPT1A), which shifts macrophages toward M1-like phenotype. Knockdown of HMGB1 can restore FAO and alleviate colitis (71). In addition, microbial metabolites, such as agmatine and cadaverine, can also modulate macrophage metabolic programming, promoting polarization toward an M2-like phenotype characterized by enhanced fatty acid oxidation (72, 73).

Notably, the metabolic switches described above are intimately connected to, yet distinct from, mitochondrial dynamics, which are the processes of mitochondrial fission, fusion, and turnover that govern organelle integrity and function. While metabolic reprogramming dictates the choice of energy substrate, mitochondrial dynamics determine the structural and functional capacity of the organelle itself. These two layers of regulation are discussed, beginning with the role of mitochondrial dynamics in UC macrophages.

**Figure 2:**
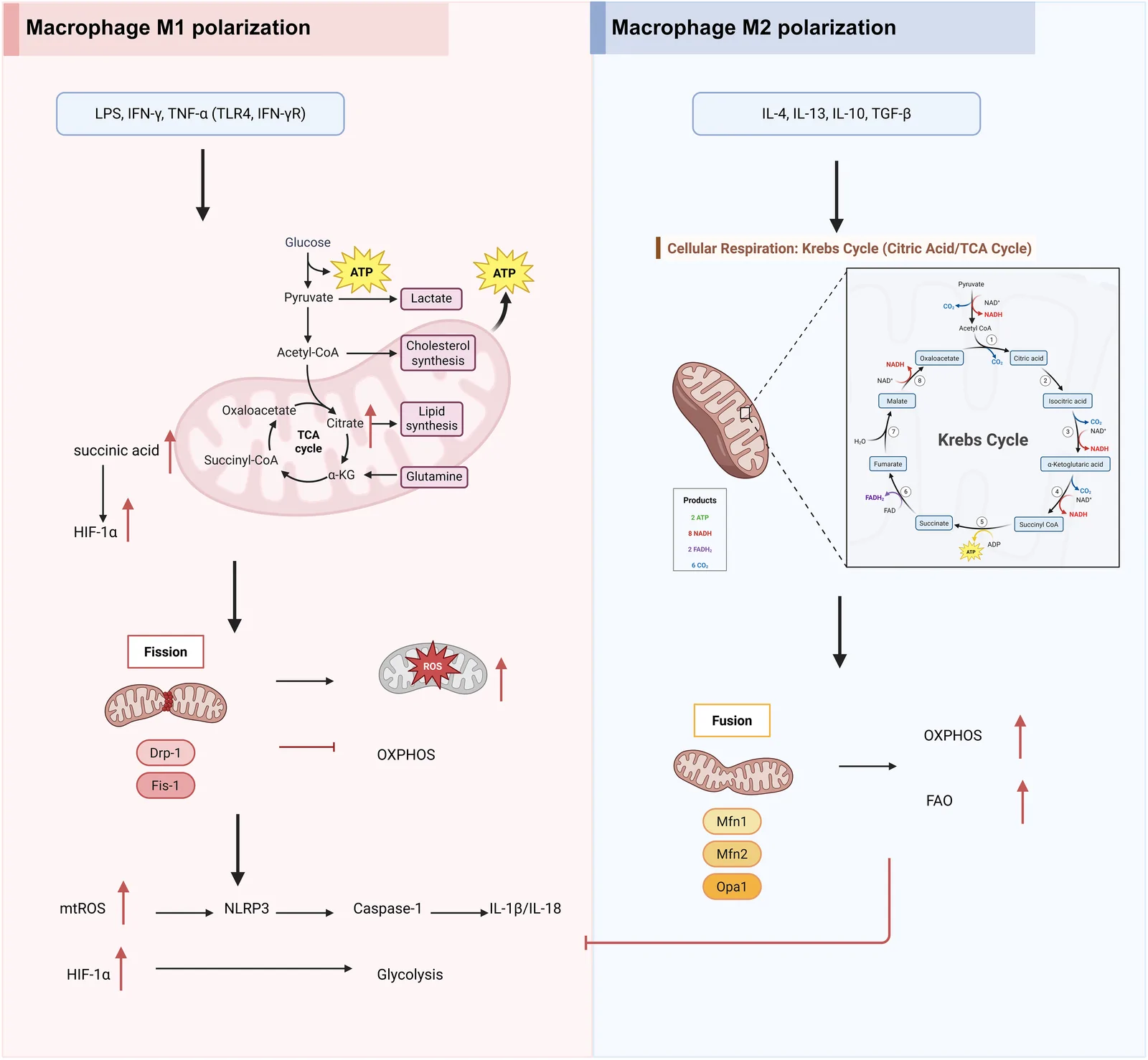
Metabolic characteristics in macrophage polarization. The M0 macrophages are exposed to various stimulating factors: the stimulating factors for M1-like type macrophage polarization (lipopolysaccharide, IFN-γ, TNF-α) and the stimulating factors for M2-like type macrophage polarization (IL-4, IL-13, IL-10, TGF-β) affecting the intracellular metabolism and mitochondrial regulatory pathways of the cells.

Several aspects of metabolic reprogramming are uniquely shaped by the intestinal microenvironment in macrophages of UC. First, the colonic epithelium generates a steep oxygen gradient which lead to constitutive stabilization of HIF-1α in tissue-resident macrophages. This primes macrophages for rapid glycolytic responses during inflammation (74). Second, the extraordinarily high concentrations of gut microbiota-derived short-chain fatty acids, such as butyrate, directly fuel macrophage oxidative metabolism and suppress pro-inflammatory gene expression through histone deacetylase inhibition in the colon (75). This metabolite-driven regulation is largely absent in extra-intestinal inflammatory conditions, where macrophage polarization is orchestrated primarily by endogenous cytokines. Third, the continuous exposure of intestinal macrophages to bacterial PAMPs, such as LPS, creates a state of tolerance in tissue-resident macrophages under homeostasis (17, 76). These intestine-specific metabolic features underscore the need to consider the local microenvironment when interpreting and targeting macrophage reprogramming in intestinal metabolic niche and macrophage adaptation of UC (**Figure 2**).

### 4.2 The mitochondrial dynamics of macrophage

Through mitochondria, ROS are generated, which regulate the inflammatory response (77). CLUH, which maintains mitochondrial stability in macrophages, is suppressed in UC. While DRP1 and ORMDL3 are activated. Suppressed expression of CLUH and activated DRP1 and ORMDL3 lead to excessive mitochondrial fission, increased ROS production, and promotion of a pro-inflammatory macrophage state (78, 79). The burst of ROS stabilizes HIF-1α through multiple pathways (80). Then, stabilized HIF-1α enters the nucleus and upregulates the expression of the glycolytic enzymes, including PKM2 (81). This forces macrophage energy metabolism to switch from OXPHOS to aerobic glycolysis. Therefore, effective clearance of mitochondrial ROS can attenuate inflammation and enable macrophages regain their reparative capacity. However, the DRP1-CLUH-ORMDL3 axis has been investigated predominantly in murine models and *in vitro* human macrophage systems. Thus, these mitochondrial dynamics pathways remains analyzed in human UC tissue.

Beyond the DRP1-CLUH-ORMDL3 axis, other key regulators of mitochondrial dynamics are increasingly implicated in macrophage function. The mitochondrial fusion and fission balance has been explored in UC. In addition to DRP1-mediated fission, mitochondrial fusion is governed by mitofusins (MFN1 and MFN2) on the outer mitochondrial membrane and by optic atrophy protein 1 (OPA1) on the inner mitochondrial membrane. MFN1/MFN2 mediate outer membrane fusion, while OPA1 regulates inner membrane fusion and cristae organization (82, 83). Furthermore, myeloid-specific deletion of MFN2, but not MFN1, is associated with impaired mitophagy, phagocytosis, and accelerated fibrosis progression (84). The balance between fission and fusion determines overall mitochondrial morphology, respiratory capacity, and susceptibility to oxidative stress. The altered mitochondrial morphology is a hallmark of activated macrophages which disruption of mitochondrial fusion and fission balance in UC.

Mitophagy, mediated by the PINK1/Parkin pathway, removes damaged mitochondria. A few studies in macrophages have demonstrated that mitophagy regulates macrophage survival, reprogramming, and inflammatory responses (85, 86). Impairment of this pathway leads to the accumulation of dysfunctional mitochondria, excessive ROS production, and sustained NLRP3 inflammasome activation (87–89). Thus, defects in mitophagy of macrophage may influence the chronic intestinal inflammation.

The mitochondrial contact site and cristae organizing system (MICOS) stabilizes cristae junctions and maintains inner mitochondrial membrane architecture. A study suggests that MICOS complex dysfunction exacerbates oxidative stress and contributes to age-dependent structural deficits in the kidney (90). This indicates that MICOS complex may have critical role in macrophage of UC.

Mitochondria also participate in intracellular calcium homeostasis. Calcium uptake into the mitochondrial matrix, mediated by the mitochondrial calcium uniporter (MCU), regulates TCA cycle enzyme activity and OXPHOS (91). Moreover, mitochondrial calcium signaling has been linked to NLRP3 inflammasome activation and metabolic reprogramming in macrophages of UC (92).

### 4.3 Intercellular interaction signals of macrophage plasticity

The macrophage plasticity is dually regulated by ROS-mediated metabolic reprogramming and direct cell-cell communication in the intestinal microenvironment of UC. DRP1-mediated mitochondrial fission produces a burst of ROS that stabilizes HIF-1α and drives PKM2-dependent glycolysis, thereby promoting an M1-like phenotype in Ly6C hi monocytes (18, 78, 80, 81). Moreover, multiple cell types within the microenvironment shape macrophage polarization through direct membrane surface interactions (93). This integration of ROS-driven metabolic programming and intercellular contact signals can influence the fate and function of macrophages in UC.

### 4.4 Transcription factors in macrophage polarization

#### 4.4.1 Transcription factors drive M1-like polarization

Transcription factors, STAT1, NF-κB, IRF5, and AP-1, drive the macrophage polarization toward the M1-like. These factors, activated by IFN-γ signaling, bind directly to promoter regions of M1-like associated genes, such as NOS2, enhancing their transcription (94). While suppressed the activity of transcription factors can promote M2-like polarization (95). There are evidence that IL1B, IL6, and CXCL8, which bind κB motifs in the regulatory regions of M1-like genes, are activated and expressed after NF-κB translocates to the nucleus upon inflammatory stimulation (96, 97). Moreover, IRF5 cooperates synergistically with NF-κB, amplifies transcription of M1-like related genes, IL12A and IL1B, while inhibiting M2-like markers such as IL10 and ARG1 (98). In addition, AP-1 promotes the expression of TNF and MMP9, stabilizes the pro-inflammatory phenotype through binding AP-1 response elements (99–101).

#### 4.4.2 Transcription factors drive M2-like polarization

Transcription factors, STAT6, PPARγ, KLF4, and STAT3, drive the macrophage polarization toward the M2-like. STAT6 which is activated by IL-4 signaling, translocates to the nucleus and binds STAT6 response elements in the promoters of M2-like marker genes, *Arg1* and *IL10*, to drive their transcription. Concurrently, it suppresses the nuclear translocation and activity of M1-like associated transcription factors, STAT1 and NF-κB (102, 103). Previous reports suggest that PPARγ promotes M2-like polarization by competing with NF-κB for genomic binding sites (104, 105). KLF4 inhibits the transcription of M1-like genes and stabilizes the M2-like. A study indicates that knockdown of KLF4 induced inflammatory cytokine expression (106). STAT3, which is activated by IL-10 and related anti-inflammatory signals, has been demonstrated to promote the expression of M2-like associated genes and reinforce M2-like macrophage identity (107).

## 5 Pathogens and metabolites in macrophage polarization

The macrophage polarization is shaped by PAMPs, DAMPs, and host-microbiota derived metabolites in UC. PAMPs, such as LPS, activate TLR4/NF-κB signaling to induce TNF-α which is a pro-inflammatory cytokine (108). While DAMPs, such as HMGB1, inhibit apoptosis and activate the NLRP3 inflammasome to sustain chronic inflammation (109). Indeed, M1-like polarization relies on glycolysis, whereas M2-like polarization is enhanced by glycolytic inhibition. The convergence of these microbial metabolites with host-derived signals is a defining feature of macrophage regulation in the gut that distinguishes IBD from other chronic inflammatory disorders (11). In rheumatoid arthritis or atherosclerosis, for example, macrophage polarization is driven primarily by endogenous cytokine networks and tissue-derived DAMPs, without the additional layer of regulation imposed by a resident microbiota (110, 111). The macrophages integrate signals from bacterial fermentation products, dietary components, and host inflammatory mediators, resulting in a metabolic and functional repertoire that is uniquely adapted to the intestinal environment in UC.

## 6 Omics analysis of macrophage diversity

Omics analysis can provide evidence of macrophage diversity in UC and colitis-associated colorectal cancer, such as single-cell sequencing, metabolomics and proteomics.

### 6.1 Transcriptomic and proteomic profiling

Transcriptomic and proteomic approaches are increasingly applied to decipher macrophage heterogeneity in UC and CRC (112–114). Integrated RNA sequencing and liquid chromatography–tandem mass spectrometry (LC-MS/MS) of intestinal tissues or isolated macrophages enable comprehensive analysis of differentially expressed mRNAs and proteins in UC (115, 116). Previous studies have identified key regulatory pathways, such as pro-inflammatory signaling *via* TLR4/NF-κB activation, using transcriptomics in colitis-associated macrophages (117). Proteomic analyses reveal how post-translational modifications (PTMs) regulate macrophage function. For example, increased phosphorylation of NF-κB p65 and MAPK p38 in macrophages directly activates pro-inflammatory pathways, as observed in gastric ulcer models (118). Indeed, the ubiquitination of IκBα, which is an inhibitor of NF-κB, is elevated in colitis-associated macrophages. This leads to its degradation by the proteasome which relieves the inhibition on NF-κB mediated by USP16 (119). Thus, the integration of transcriptome and proteome sequencing provides a powerful strategy for identifying key genes and proteins that orchestrate macrophage polarization in inflammation of UC and colitis-associated colorectal cancer.

### 6.2 Metabolomics

Intestinal macrophages are key regulators of mucosal inflammation. Numerous studies suggest that metabolomics can analyze the small-molecule metabolites, including carbohydrates, lipids, amino acids, and nucleotide derivatives, which can clarify the role of metabolic reprogramming in macrophage polarization (11, 120). Following metabolite extraction from enriched macrophage populations, differential metabolites and their associated pathways are identified through functional correlation analysis. Compared with traditional transcriptomics, metabolomics can directly characterize the glycolysis dependence of M1-like macrophages and the FAO deficiency of M2-like macrophages in UC. There is evidence showing that LPS-induced M1-like polarization drives extensive metabolic reprogramming, such as enhanced aerobic glycolysis and disruption of the tricarboxylic acid cycle (80). Notably, conventional metabolomics reflects the average metabolic state of macrophage populations. The inability to distinguish monocyte-derived from tissue-resident subsets is a limitation of conventional metabolomics, which can be addressed by integrating metabolomics with single-cell transcriptomics.

### 6.3 Single-cell RNA sequencing

The scRNA-seq enables high-resolution analysis of gene expression patterns in intestinal macrophages from patients with colitis and CRC. The scRNA-seq reveals cellular heterogeneity, functional states, and spatial distributions (121). The intestinal immune cells are isolated through fluorescence-activated cell sorting (FACS) to enrich macrophage populations from UC or CRC tissues, followed by scRNA-seq library preparation and sequencing. This appro46ach has been widely applied to define distinct macrophage subtypes that extend far beyond the traditional M1/M2 framework. The scRNA-seq data showed several macrophage populations with unique transcriptomic signatures in UC. The *FCGR3A*^+^ (CD16^+^) inflammatory macrophages which express high levels of *IL1B*, *TNF*, and *TREM1* are abundant in active disease. The *SELENOP*^+^ tissue-resident macrophages are present in both healthy and inflamed tissues and express *CD163* and *C1QA.* The *IL1RN*^+^ macrophages emerge during the resolution phase and are associated with epithelial repair (21, 36). These subsets co-exist within the same tissue and can dynamically transition between states. Moreover, the characteristic gene expression profiles are analyzed in IBD (122). In addition, scRNA-seq data can be integrated with spatial transcriptomic methods to reconstruct the anatomical localization of macrophage subsets within intestinal tissues (123). Compared with conventional sequencing, scRNA-seq provides unprecedented resolution to dissect macrophage heterogeneity, track dynamic phenotypic changes, and decipher cell-cell communication networks within the intestinal microenvironment.

## 7 The role of macrophages in clinical applications

As core regulatory cells of intestinal innate and adaptive immunity, macrophages influence UC. Targeted to macrophage interventions is effect in clinical study (**Table 2**).

**Table 2:** Clinical development status of macrophage-targeted interventions in UC.

Intervention	Mechanism	Development stage in UC	Reference
Infliximab (anti-TNF)	Neutralize soluble/membrane TNF-α	Approved	(124)
Tofacitinib (JAK inhibitor)	Inhibit JAK-STAT signaling	Approved	(125)
Ustekinumab (anti-IL-12/23)	Block IL-12/23 p40	Approved	(^1^, 126)
Cenicriviroc (CCR2/CCR5 antagonist)	Regulatory T cells	Experimental	(127)
CAR-macrophage therapy	Engineered M2-polarized macrophages	Preclinical	(^2^)
Nanoparticle-mediated targeting	Drug delivery to macrophages	Preclinical	(^3^)
PPAR-γ agonists (rosiglitazone)	Induce M2 polarization	Preclinical	(^4^)
Probiotic-derived SCFAs	Suppress NF-κB, promote FAO	Preclinical/experimental	(^5^, 75)

^1^ Sands BE, Sandborn WJ, Panaccione R, O'Brien CD, Zhang H, Johanns J, et al. Ustekinumab as Induction and Maintenance Therapy for Ulcerative Colitis. *N Engl J Med* (2019) 381(13):1201-14. doi: 10.1056/NEJMoa1900750.

^2^ Cao Q, Wang Y, Chen J, Wang R, Chen T, Gloss B, et al. Targeting Inflammation with Chimeric Antigen Receptor Macrophages Using a Signal Switch. *Nat Biomed Eng* (2025) 9(9):1502-16. Epub 20250507. doi: 10.1038/s41551-025-01387-8.

^3^ Pi F, Yang F, Zhan Z, Xue Q, Wu Q, Yuan Z, et al. Oral Delivery of Ebselen Nanoparticle Modulating Macrophage Polarization for Ulcerative Colitis Treatment. *Cell Biomaterials* (2026):100418. doi: 10.1016/j.celbio.2026.100418.

^4^ Dubuquoy L, Jansson EA, Deeb S, Rakotobe S, Karoui M, Colombel JF, et al. Impaired Expression of Peroxisome Proliferator-Activated Receptor Gamma in Ulcerative Colitis. *Gastroenterology* (2003) 124(5):1265-76. doi: 10.1016/s0016-5085(03)00271-3.

^5^ Yu S, Zhang M, Dou Z, Tian B, Lu J. Gut Microbiota Metabolites in the Immunoregulation of Enteritis: Research Progress. *Front Immunol* (2025) 16:1706472. Epub 20251208. doi: 10.3389/fimmu.2025.1706472.

### 7.1 Therapeutic modulation of macrophage polarization

Currently, modulating macrophage polarization is the aim of therapeutic strategies in UC. These include promoting M2-like anti-inflammatory phenotypes or suppressing M1-like activation. Administration of exogenous IL-10 can drive M2-like differentiation and suppress pro-inflammatory cytokine release. Similarly, PPAR-γ agonists (e.g., rosiglitazone) and berberine have been shown to induce M2-like polarization and exert intestinal protective effects (47, 128, 129). These induce M2-like polarization and exert intestinal protective effects. Conversely, M1-like inhibition can be achieved through the JAK inhibitor (tofacitinib). This modulates macrophage polarization (130). Collectively, these data indicate that macrophage polarization toward the M2-like phenotype represents a viable therapeutic strategy in UC. It is noteworthy that a few macrophage-targeted therapies show differential efficacy between UC and CD. For example, tofacitinib (JAK inhibitor) is approved for UC (125), however, it has not demonstrated consistent efficacy in CD (131). These clinical observations may reflect underlying differences in the dominant macrophage polarization states and cytokine networks in UC versus CD.

### 7.2 Suppress excessive macrophage activation

TNF-α and IL-1β, which are pro-inflammatory cytokines, are the key mediators of macrophage polarization in UC. Their therapeutic blockade represents a clinically validated strategy to mitigate macrophage-driven inflammation. Monoclonal antibodies, such as infliximab, have a role in neutralizing soluble and membrane-bound TNF-α. Then, preventing its interaction with macrophage and T cell receptors, thereby attenuating downstream inflammatory signaling (124, 132). In addition, a study suggests that anti-IL-1β therapy reduces macrophage infiltration and limits tissue damage (133). Moreover, the intracellular approach involves direct inhibition of signaling cascades that sustain macrophage activation, such as suppression of the MAPK pathway. This disrupts the transcription and translation of pro-inflammatory cytokines, including TNF-α and IL-6 (134). These strategies suggest that targeting activated macrophages has the therapeutic potential at extracellular and intracellular levels.

### 7.3 Target macrophage recruitment

To disrupt the aberrant recruitment of monocytes and macrophages to inflamed intestinal tissue is a therapeutic strategy in UC. Cenicriviroc, a dual antagonist of CCR2 and CCR5, reduces the accumulation and effector functions of pro-inflammatory immune cells and effectively blocks pathological macrophage recruitment (127). A previous study indicated that macrophages highly express CX3CR1, a chemokine receptor that mediates adhesion to epithelial cells *via* its ligand CX3CL1 in the intestinal lamina propria. Modulating CX3CR1 activity can diminish macrophage retention within the intestinal mucosa to control inflammation (135). These approaches suggest that interfering with chemokine-mediated trafficking and localization of macrophages has a therapeutic effect in UC.

### 7.4 Macrophage-directed cellular therapy

To modulate intestinal inflammation, a cell-based therapeutic strategy has emerged. A study showed that *in vitro*-generated M2-like polarized macrophages which are derived from patient monocytes can be administered to exert anti-inflammatory and tissue-reparative effects (17, 42, 136). In addition, chimeric antigen receptor (CAR)-macrophage engineering can enhance local accumulation and polarization. CARs designed against antigens enriched in the inflammatory microenvironment, such as TNF-α receptors or TLR4, can direct macrophages to sites of intestinal inflammation. Furthermore, CAR signaling can be engineered to suppress pro-inflammatory functions and promote an M2-like reparative phenotype in IBD (137, 138).

These advances reveal that combining macrophage-targeted interventions, through polarization control, recruitment blockade, or adoptive cell transfer, into integrated immunotherapy holds promise for UC and colitis-associated cancer.

### 7.5 Emerging macrophage-targeted strategies

Several emerging strategies have established for the future of macrophage-targeted therapy in UC. Nanoparticles can be engineered to deliver drugs, siRNAs, or metabolites specifically to intestinal macrophages. For example, PLGA nanoparticles loaded with andrographolide agonists can accumulate in colonic macrophages (139). Epigenetic modifications, including histone acetylation and DNA methylation, play critical roles in establishing and maintaining macrophage polarization states. HDAC inhibitors promote M2-like polarization by relaxing chromatin (140, 141). Bromodomain and extra terminal domain (BET) bromodomain inhibitors can suppress pro-inflammatory gene transcription (142, 143). A growing number of clinical trials are specifically designed to evaluate macrophage-targeted interventions in IBD. These include trials of CSF1R inhibitors that deplete pro-inflammatory macrophages (144).

## 8 Discussion and conclusion

The sustained release of ROS and inflammatory mediators by macrophages and neutrophils perpetuates the inflammatory microenvironment in UC (145). Macrophages have been demonstrated to exhibit a functional plasticity which adapts their phenotype in response to local signals. Previous studies suggested that M1-like macrophages can exert anti-tumor effects through antigen presentation and activation of cytotoxic T cells. While sustained inflammation promotes M1-like toward M2-like polarization which is involved tumor progression (146, 147). Evidence has shown that M2-like macrophages foster an immunosuppressive niche *via* secretion of IL-10 and TGF-β by inhibiting the activity of T cells and NK cells (148, 149). Moreover, M2-like macrophages may also express PD-L1 which engages PD-1 on T cells that inducing functional exhaustion (150). Thus, modulating macrophage polarization is crucial for intercepting inflammation-driven tumorigenesis. However, it is unclear whether the metabolic reprogramming observed in UC macrophages is a cause or consequence of inflammation. The relative contributions of embryo-derived tissue-resident macrophages versus monocyte-derived macrophages to tissue repair in human UC have not been directly assessed. The field is moving beyond the M1/M2 paradigm toward a more nuanced understanding of macrophage states defined by single-cell transcriptomic signatures.

Furthermore, macrophages shape intestinal immunity through coordinated interactions with other immune populations. Numerous studies have suggested that macrophages secrete CXCL8, CXCL1, and CXCL2 to recruit neutrophils to inflammatory foci. Then, neutrophils release NETs (151–153). Subsequently, macrophages clear pathogenic debris and apoptotic neutrophils that contribute to inflammation resolution. Additionally, macrophages enhance T cell responses through antigen presentation and cytokine secretion (154, 155). Moreover, macrophages release IL-12 and IL-18 which enable synergistic activation of NK cells. The activated NK cells promote the elimination of infected cells. These interactions demonstrate the central role of macrophages in coordinating innate and adaptive immunity in UC and associated cancer.

Targeting macrophages represents a promising translational strategy for UC treatment. Evidence has shown that probiotic-derived metabolites, such as short-chain fatty acids, can suppress NF-κB activation in macrophages by reducing IL-6 and TNF-α production (156, 157). Previous study suggested that IL-22 promotes anti-inflammatory macrophage function through induction of TGF-β and IL-10 (135, 158). In addition, colony-stimulating factor 1 receptor (CSF-1R) inhibitors deplete tumor-associated macrophages (TAMs) *via* apoptosis (159). Moreover, IFN-γ can reprogram TAMs toward an M1-like anti-tumorigenic phenotype (160, 161).

The inflammation is typically continuous and confined to the mucosa and superficial submucosa, creating a microenvironment characterized by severe epithelial erosion, hypoxic stress, and high local concentrations of ROS in UC (2, 19, 74). Accordingly, the selective depletion of embryo-derived tissue-resident macrophages through oxidative stress which mediated by SOD2 deficiency has been specifically demonstrated in UC lesions (19). This phenomenon may be less prominent in CD. The transmural inflammation features prominent macrophage-rich granulomas and extensive fibrosis in CD (162). CCL2-driven accumulation of monocyte-derived macrophages in the deep intestinal wall is a hallmark of CD (17). Moreover, single-cell transcriptomic studies have identified abundance of *FCGR3A*^+^ inflammatory macrophages in UC, whereas *TREM1*^+^ and fibrosis-associated macrophage subsets are more characteristic of CD (21, 36, 163). The anti-TNF agents are effective in both UC and CD (124, 164). The anti-IL-12/23 therapy (ustekinumab) has shown robust efficacy in CD (126), while tofacitinib is predominantly approved for UC (125). These observations suggest that macrophage polarization pathways may be differentially engaged in UC versus CD.

Future research should leverage multi-omics approaches, such as single-cell transcriptomics, spatial proteomics, and metabolomics. These approaches construct a comprehensive atlas of macrophage states across the full spectrum of UC. It is essential to identify targetable metabolic checkpoints, particularly those governing the switch between glycolysis and fatty acid oxidation or controlling mitochondrial fission/fusion dynamics. Furthermore, clinical translation have ability to validate of macrophage-targeted interventions in randomized trials, with careful assessment of their effects on both inflammation and mucosal repair. The application of single-cell and spatial technologies in clinical trials offers an unprecedented opportunity to monitor macrophage reprogramming in real time and to develop companion biomarkers for patient stratification.

## References

[1] Kobayashi T Siegmund B Le Berre C Wei SC Ferrante M Shen B . Ulcerative colitis. Nat Rev Dis Primers. (2020) 6:74. doi: 10.1007/978-4-431-56018-0_16 32913180

[2] Ordás I Eckmann L Talamini M Baumgart DC Sandborn WJ . Ulcerative colitis. Lancet. (2012) 380:1606–19. doi: 10.1016/S0140-6736(12)60150-0 22914296

[3] Mass E Nimmerjahn F Kierdorf K Schlitzer A . Tissue-specific macrophages: how they develop and choreograph tissue biology. Nat Rev Immunol. (2023) 23:563–79. doi: 10.1038/s41577-023-00848-y 36922638 PMC10017071

[4] Murray PJ Wynn TA . Protective and pathogenic functions of macrophage subsets. Nat Rev Immunol. (2011) 11:723–37. doi: 10.1038/nri3073 21997792 PMC3422549

[5] Moldoveanu AC Diculescu M Braticevici CF . Cytokines in inflammatory bowel disease. Romanian J Internal Med. (2015) 53:118–27. doi: 10.1515/rjim-2015-0016 26402980

[6] Zhang J Zhao Y Hou T Zeng H Kalambhe D Wang B . Macrophage-based nanotherapeutic strategies in ulcerative colitis. J Controlled Release. (2020) 320:363–80. doi: 10.1016/j.jconrel.2020.01.047 32001299

[7] Silverstein AM . Ilya Metchnikoff, the phagocytic theory, and how things often work in science. J Leukoc Biol. (2011) 90:409–10. doi: 10.1189/jlb.0511234 21880643

[8] Kloc M Kubiak JZ . The role of monocytes and macrophages in homeostasis and disease and novel avenues for putative treatments. Int J Mol Sci. (2021) 22(9):4927. doi: 10.3390/ijms22094927 34066450 PMC8125502

[9] Shi C Pamer EG . Monocyte recruitment during infection and inflammation. Nat Rev Immunol. (2011) 11:762–74. doi: 10.1038/nri3070 21984070 PMC3947780

[10] Ginhoux F Guilliams M . Tissue-resident macrophage ontogeny and homeostasis. Immunity. (2016) 44:439–49. doi: 10.1016/j.immuni.2016.02.024 26982352

[11] O'Neill LA Kishton RJ Rathmell J . A guide to immunometabolism for immunologists. Nat Rev Immunol. (2016) 16:553–65. doi: 10.1038/nri.2016.70 PMC5001910 27396447

[12] Zigmond E Varol C Farache J Elmaliah E Satpathy AT Friedlander G . Ly6C hi monocytes in the inflamed colon give rise to proinflammatory effector cells and migratory antigen-presenting cells. Immunity. (2012) 37:1076–90. doi: 10.1016/j.immuni.2012.08.026 23219392

[13] Shaw TN Houston SA Wemyss K Bridgeman HM Barbera TA Zangerle-Murray T . Tissue-resident macrophages in the intestine are long lived and defined by Tim-4 and CD4 expression. J Exp Med. (2018) 215:1507–18. doi: 10.1084/jem.20180019 29789388 PMC5987925

[14] Peng M Li N Wang H Li Y Liu H Luo Y . Macrophages: subtypes, distribution, polarization, immunomodulatory functions, and therapeutics. Medcomm (2020). (2025) 6:e70304. doi: 10.1002/mco2.70304 40717900 PMC12290311

[15] Zhang M Li X Zhang Q Yang J Liu G . Roles of macrophages on ulcerative colitis and colitis-associated colorectal cancer. Front Immunol. (2023) 14:1103617. doi: 10.3389/fimmu.2023.1103617 37006260 PMC10062481

[16] Bain CC Schridde A . Origin, differentiation, and function of intestinal macrophages. Front Immunol. (2018) 9:2733. doi: 10.3389/fimmu.2018.02733 30538701 PMC6277706

[17] Bain CC Mowat AM . Macrophages in intestinal homeostasis and inflammation. Immunol Rev. (2014) 260:102–17. doi: 10.1111/imr.12192 24942685 PMC4141699

[18] Bain CC Scott CL Uronen-Hansson H Gudjonsson S Jansson O Grip O . Resident and pro-inflammatory macrophages in the colon represent alternative context-dependent fates of the same Ly6Chi monocyte precursors. Mucosal Immunol. (2013) 6:498–510. doi: 10.1038/mi.2012.89 22990622 PMC3629381

[19] Du J Zhang J Wang L Wang X Zhao Y Lu J . Selective oxidative protection leads to tissue topological changes orchestrated by macrophage during ulcerative colitis. Nat Commun. (2023) 14:3675. doi: 10.1038/s41467-023-39173-2 37344477 PMC10284839

[20] Hegarty LM Jones GR Biram A Adams CE Gentek RM Ho GT . Tissue resident colonic macrophages persist through acute inflammation and adapt to aid tissue repair. Mucosal Immunol. (2026) 19:1624–35. doi: 10.1101/2025.09.19.677333 41248849 PMC7619198

[21] Martin JC Chang C Boschetti G Ungaro R Giri M Grout JA . Single-cell analysis of Crohn's disease lesions identifies a pathogenic cellular module associated with resistance to anti-TNF therapy. Cell. (2019) 178:1493–1508.e20. doi: 10.1016/j.cell.2019.08.008 31474370 PMC7060942

[22] Pedersen G . Development, validation and implementation of an *in vitro* model for the study of metabolic and immune function in normal and inflamed human colonic epithelium. Dan Med J. (2015) 62:B4973. 25557335

[23] Shapouri-Moghaddam A Mohammadian S Vazini H Taghadosi M Esmaeili SA Mardani F . Macrophage plasticity, polarization, and function in health and disease. J Cell Physiol. (2018) 233:6425–40. doi: 10.1002/jcp.26429 29319160 PMC13482560

[24] Zhang J Liu X Wan C Liu Y Wang Y Meng C . NLRP3 inflammasome mediates M1 macrophage polarization and IL-1β production in inflammatory root resorption. J Clin Periodontol. (2020) 47:451–60. doi: 10.1111/jcpe.13258 31976565

[25] Liu C Gong Q Liu W Zhao Y Yan X Yang T . Berberine-loaded PLGA nanoparticles alleviate ulcerative colitis by targeting IL-6/IL-6R axis. J Transl Med. (2024) 22:963. doi: 10.1186/s12967-024-05682-x 39448992 PMC11515557

[26] Chang C Chen G Wu W Chen D Chen S Gao J . Exogenous IL-25 ameliorates airway neutrophilia via suppressing macrophage M1 polarization and the expression of IL-12 and IL-23 in asthma. Respir Res. (2023) 24:260. doi: 10.1186/s12931-023-02557-5 37898756 PMC10613395

[27] Wu J Pang X Yang X Zhang M Chen B Fan H . M1 macrophages induce PD-L1(hi) cell-led collective invasion in HPV-positive head and neck squamous cell carcinoma via TNF-α/CDK4/UPS14. J Immunother Cancer. (2023) 11(12):e007670. doi: 10.1016/b978-0-12-817868-3.00015-9 38148114 PMC10753854

[28] Wu K Yuan Y Yu H Dai X Wang S Sun Z . The gut microbial metabolite trimethylamine N-oxide aggravates GVHD by inducing M1 macrophage polarization in mice. Blood. (2020) 136:501–15. doi: 10.1182/blood.2019003990 32291445 PMC7378459

[29] Li S Chen Z Wang M Rao Y Yang F Liu M . L-arginine-modified selenium nanozymes targeting M1 macrophages for oral treatment of ulcerative colitis. Small. (2025) 21:e2408205. doi: 10.1002/smll.202408205 39763139

[30] Malik JA Affan Khan M Lamba T Adeel Zafar M Nanda S Owais M . Immunosuppressive effects of morphine on macrophage polarization and function. Eur J Pharmacol. (2024) 975:176637. doi: 10.1016/j.ejphar.2024.176637 38729416

[31] Wang H Huang T Ma Y . Mechanisms and therapeutic strategies of macrophages and neutrophils inducing ulcerative colitis progression. Front Immunol. (2025) 16:1615340. doi: 10.3389/fimmu.2025.1615340 40948760 PMC12425758

[32] Zhang K Guo J Yan W Xu L . Macrophage polarization in inflammatory bowel disease. Cell Commun Signal. (2023) 21:367. doi: 10.1186/s12964-023-01386-9 38129886 PMC10734116

[33] Yao Y Xu T Li X Shi X Wu H Zhang Z . Selenoprotein S maintains intestinal homeostasis in ulcerative colitis by inhibiting necroptosis of colonic epithelial cells through modulation of macrophage polarization. Theranostics. (2024) 14:5903–25. doi: 10.7150/thno.97005 39346531 PMC11426251

[34] Razi S Yaghmoorian Khojini J Kargarijam F Panahi S Tahershamsi Z Tajbakhsh A . Macrophage efferocytosis in health and disease. Cell Biochem Funct. (2023) 41:152–65. doi: 10.1002/cbf.3780 36794573

[35] Yang R Liao Y Wang L He P Hu Y Yuan D . Exosomes derived from M2b macrophages attenuate DSS-induced colitis. Front Immunol. (2019) 10:2346. doi: 10.3389/fimmu.2019.02346 31749791 PMC6843072

[36] Smillie CS Biton M Ordovas-Montanes J Sullivan KM Burgin G Graham DB . Intra- and inter-cellular rewiring of the human colon during ulcerative colitis. Cell. (2019) 178:714–730.e22. doi: 10.1016/j.cell.2019.06.029 31348891 PMC6662628

[37] Saurabh NK Khan MM Kirabo A . A future avenue of treatment ulcerative colitis targeting macrophage polarization: a phytochemical application. Crohns Colitis 360. (2024) 6:otae070. doi: 10.1093/crocol/otae070 39668979 PMC11635166

[38] Chapuy L Bsat M Sarkizova S Rubio M Therrien A Wassef E . Two distinct colonic CD14(+) subsets characterized by single-cell RNA profiling in Crohn's disease. Mucosal Immunol. (2019) 12:703–19. doi: 10.1038/s41385-018-0126-0 30670762

[39] De Schepper S Verheijden S Aguilera-Lizarraga J Viola MF Boesmans W Stakenborg N . Self-maintaining gut macrophages are essential for intestinal homeostasis. Cell. (2018) 175:400–415.e13. doi: 10.1016/j.cell.2018.07.048 30173915

[40] Muller PA Koscsó B Rajani GM Stevanovic K Berres ML Hashimoto D . Crosstalk between muscularis macrophages and enteric neurons regulates gastrointestinal motility. Cell. (2014) 158:300–13. doi: 10.1016/j.cell.2014.08.002 25036630 PMC4149228

[41] Zhang D Tao P Li J Zhou X Qu H Li Y . Targeting PKM2-dependent glycolysis reprogrammes monocytes into Cadm1+ macrophages to promote mucosal repair and attenuate colitis progression. (2026) gutjnl-2025-337344. doi: 10.1136/gutjnl-2025-337344 41986135

[42] Na YR Stakenborg M Seok SH Matteoli G . Macrophages in intestinal inflammation and resolution: a potential therapeutic target in IBD. Nat Rev Gastroenterol Hepatol. (2019) 16:531–43. doi: 10.1038/s41575-019-0172-4 31312042

[43] Ivashkiv LB . IFNγ: signalling, epigenetics and roles in immunity, metabolism, disease and cancer immunotherapy. Nat Rev Immunol. (2018) 18:545–58. doi: 10.1038/s41577-018-0029-z 29921905 PMC6340644

[44] Murray PJ Allen JE Biswas SK Fisher EA Gilroy DW Goerdt S . Macrophage activation and polarization: nomenclature and experimental guidelines. Immunity. (2014) 41:14–20. doi: 10.1016/j.immuni.2014.07.009 25035950 PMC4123412

[45] Wynn TA Vannella KM . Macrophages in tissue repair, regeneration, and fibrosis. Immunity. (2016) 44:450–62. doi: 10.1016/j.immuni.2016.02.015 26982353 PMC4794754

[46] Wynn TA . IL-13 effector functions. Annu Rev Immunol. (2003) 21:425–56. doi: 10.1146/annurev.immunol.21.120601.141142 12615888

[47] Murray PJ . The JAK-STAT signaling pathway: input and output integration. J Immunol. (2007) 178:2623–9. doi: 10.4049/jimmunol.178.5.2623 17312100

[48] Li MO Wan YY Sanjabi S Robertson AK Flavell RA . Transforming growth factor-beta regulation of immune responses. Annu Rev Immunol. (2006) 24:99–146. doi: 10.1146/annurev.immunol.24.021605.090737 16551245

[49] Zimmerman NP Vongsa RA Wendt MK Dwinell MB . Chemokines and chemokine receptors in mucosal homeostasis at the intestinal epithelial barrier in inflammatory bowel disease. Inflammatory Bowel Dis. (2008) 14:1000–11. doi: 10.1002/ibd.20480 18452220 PMC4114077

[50] Döring Y Pawig L Weber C Noels H . The CXCL12/CXCR4 chemokine ligand/receptor axis in cardiovascular disease. Front Physiol. (2014) 5:212. doi: 10.3389/fphys.2014.00212 24966838 PMC4052746

[51] Bazan JF Bacon KB Hardiman G Wang W Soo K Rossi D . A new class of membrane-bound chemokine with a CX3C motif. Nature. (1997) 385:640–4. doi: 10.1038/385640a0 9024663

[52] Kvedaraite E Lourda M Mouratidou N Düking T Padhi A Moll K . Intestinal stroma guides monocyte differentiation to macrophages through GM-CSF. Nat Commun. (2024) 15:1752. doi: 10.1038/s41467-024-46076-3 38409190 PMC10897309

[53] Zwicker S Martinez GL Bosma M Gerling M Clark R Majster M . Interleukin 34: a new modulator of human and experimental inflammatory bowel disease. Clin Sci (Lond). (2015) 129:281–90. doi: 10.1042/cs20150176 25896238 PMC4557398

[54] Zoroddu S Di Lorenzo B Paliogiannis P Mangoni AA Carru C Zinellu A . Vascular endothelial growth factor in inflammatory bowel disease: a systematic review and meta-analysis. Eur J Clin Invest. (2025) 55:e14361. doi: 10.1111/eci.14361 39545600 PMC11810564

[55] Beck PL Podolsky DK . Growth factors in inflammatory bowel disease. Inflammatory Bowel Dis. (1999) 5:44–60. doi: 10.1002/ibd.3780050108 10028449

[56] Luzentales-Simpson M Pang YCF Zhang A Sousa JA Sly LM . Vedolizumab: potential mechanisms of action for reducing pathological inflammation in inflammatory bowel diseases. Front Cell Dev Biol. (2021) 9:612830. doi: 10.3389/fcell.2021.612830 33614645 PMC7887288

[57] Cuzzocrea S Rossi A Mazzon E Di Paola R Genovese T Muià C . 5-Lipoxygenase modulates colitis through the regulation of adhesion molecule expression and neutrophil migration. Lab Invest. (2005) 85:808–22. doi: 10.1038/labinvest.3700276 15821759

[58] Mori N Horie Y Gerritsen ME Anderson DC Granger DN . Anti-inflammatory drugs and endothelial cell adhesion molecule expression in murine vascular beds. Gut. (1999) 44:186–95. doi: 10.1136/gut.44.2.186 9895377 PMC1727380

[59] Barclay AN . Membrane proteins with immunoglobulin-like domains--a master superfamily of interaction molecules. Semin Immunol. (2003) 15:215–23. doi: 10.1016/s1044-5323(03)00047-2 14690046

[60] Iwasaki A Medzhitov R . Toll-like receptor control of the adaptive immune responses. Nat Immunol. (2004) 5:987–95. doi: 10.1038/ni1112 15454922

[61] Banchereau J Steinman RM . Dendritic cells and the control of immunity. Nature. (1998) 392:245–52. doi: 10.1038/32588 9521319

[62] Grewal IS Flavell RA . CD40 and CD154 in cell-mediated immunity. Annu Rev Immunol. (1998) 16:111–35. doi: 10.1146/annurev.immunol.16.1.111 9597126

[63] Vignali DA Collison LW Workman CJ . How regulatory T cells work. Nat Rev Immunol. (2008) 8:523–32. doi: 10.1038/nri2343 18566595 PMC2665249

[64] Soehnlein O Lindbom L . Phagocyte partnership during the onset and resolution of inflammation. Nat Rev Immunol. (2010) 10:427–39. doi: 10.1038/nri2779 20498669

[65] Warnatsch A Ioannou M Wang Q Papayannopoulos V . Inflammation. Neutrophil extracellular traps license macrophages for cytokine production in atherosclerosis. Science. (2015) 349:316–20. doi: 10.1126/science.aaa8064 26185250 PMC4854322

[66] De Schepper S . Self-maintaining gut macrophages are essential for intestinal homeostasis. Cell. (2019) 176:676. doi: 10.1016/j.cell.2018.07.048 30682373

[67] Klos A Tenner AJ Johswich KO Ager RR Reis ES Köhl J . The role of the anaphylatoxins in health and disease. Mol Immunol. (2009) 46:2753–66. doi: 10.1016/j.molimm.2009.04.027 19477527 PMC2725201

[68] Ricklin D Hajishengallis G Yang K Lambris JD . Complement: a key system for immune surveillance and homeostasis. Nat Immunol. (2010) 11:785–97. doi: 10.1038/ni.1923 20720586 PMC2924908

[69] Liszewski MK Kolev M Le Friec G Leung M Bertram PG Fara AF . Intracellular complement activation sustains T cell homeostasis and mediates effector differentiation. Immunity. (2013) 39:1143–57. doi: 10.1016/j.immuni.2013.10.018 24315997 PMC3865363

[70] Arbore G Kemper C Kolev M . Intracellular complement - the complosome - in immune cell regulation. Mol Immunol. (2017) 89:2–9. doi: 10.1016/j.molimm.2017.05.012 28601357 PMC7112704

[71] Wang F Luo L Wu Z Wan L Li F Wen Z . HMGB1 modulates macrophage metabolism and polarization in ulcerative colitis by inhibiting Cpt1a expression. Front Biosci (Landmark Ed). (2024) 29:387. doi: 10.31083/j.fbl2911387 39614449

[72] Zhang S Sun Z Li Y Du X Qian K Yang L . Agmatine attenuates the severity of immunometabolic disorders by suppressing macrophage polarization: an *in vivo* study using an ulcerative colitis mouse model. BioMed Pharmacother. (2024) 180:117549. doi: 10.1016/j.biopha.2024.117549 39413617

[73] de Oliveira Formiga R Li Q Zhao Y Campos Ribeiro MA Guarino-Vignon P Fatouh R . Immunometabolic reprogramming of macrophages by gut microbiota-derived cadaverine controls colon inflammation. Cell Host Microbe. (2025) 33:1855–1872.e10. doi: 10.1016/j.chom.2025.09.009 41033313

[74] Colgan SP Taylor CT . Hypoxia: an alarm signal during intestinal inflammation. Nat Rev Gastroenterol Hepatol. (2010) 7:281–7. doi: 10.1038/nrgastro.2010.39 20368740 PMC4077542

[75] Schulthess J Pandey S Capitani M Rue-Albrecht KC Arnold I Franchini F . The short chain fatty acid butyrate imprints an antimicrobial program in macrophages. Immunity. (2019) 50:432–445.e7. doi: 10.1016/j.immuni.2018.12.018 30683619 PMC6382411

[76] Smythies LE Sellers M Clements RH Mosteller-Barnum M Meng G Benjamin WH . Human intestinal macrophages display profound inflammatory anergy despite avid phagocytic and bacteriocidal activity. J Clin Invest. (2005) 115:66–75. doi: 10.1172/jci200519229 15630445 PMC539188

[77] West AP Shadel GS Ghosh S . Mitochondria in innate immune responses. Nat Rev Immunol. (2011) 11:389–402. doi: 10.1038/nri2975 21597473 PMC4281487

[78] Khan S Raj D Sahu S Naseer A Singh NC Kumari S . CLUH functions as a negative regulator of inflammation in human macrophages and determines ulcerative colitis pathogenesis. JCI Insight. (2023) 8(11):e161096. doi: 10.1172/jci.insight.161096 37140992 PMC10393232

[79] Sharma J Khan S Singh NC Sahu S Raj D Prakash S . ORMDL3 regulates NLRP3 inflammasome activation by maintaining ER-mitochondria contacts in human macrophages and dictates ulcerative colitis patient outcome. J Biol Chem. (2024) 300:107120. doi: 10.1016/j.jbc.2024.107120 38417794 PMC11065740

[80] Tannahill GM Curtis AM Adamik J Palsson-McDermott EM McGettrick AF Goel G . Succinate is an inflammatory signal that induces IL-1β through HIF-1α. Nature. (2013) 496:238–42. doi: 10.1038/nature11986 PMC4031686 23535595

[81] Palsson-McDermott EM Curtis AM Goel G Lauterbach MA Sheedy FJ Gleeson LE . Pyruvate kinase M2 regulates Hif-1α activity and IL-1β induction and is a critical determinant of the warburg effect in LPS-activated macrophages. Cell Metab. (2015) 21:65–80. doi: 10.1016/j.cmet.2014.12.005 25565206 PMC5198835

[82] Chan DC . Mitochondrial fusion and fission in mammals. Annu Rev Cell Dev Biol. (2006) 22:79–99. doi: 10.1146/annurev.cellbio.22.010305.104638 16704336

[83] Westermann B . Mitochondrial fusion and fission in cell life and death. Nat Rev Mol Cell Biol. (2010) 11:872–84. doi: 10.1038/nrm3013 21102612

[84] Bhatia D Capili A Nakahira K Muthukumar T Torres LK Choi AMK . Conditional deletion of myeloid-specific mitofusin 2 but not mitofusin 1 promotes kidney fibrosis. Kidney Int. (2022) 101:963–86. doi: 10.1681/asn.20203110s1229d PMC9038692 35227692

[85] Larson-Casey JL Deshane JS Ryan AJ Thannickal VJ Carter AB . Macrophage Akt1 kinase-mediated mitophagy modulates apoptosis resistance and pulmonary fibrosis. Immunity. (2016) 44:582–96. doi: 10.1016/j.immuni.2016.01.001 26921108 PMC4794358

[86] Bhatia D Chung KP Nakahira K Patino E Rice MC Torres LK . Mitophagy-dependent macrophage reprogramming protects against kidney fibrosis. JCI Insight. (2019) 4(23):e132826. doi: 10.1172/jci.insight.132826 31639106 PMC6962025

[87] Youle RJ Narendra DP . Mechanisms of mitophagy. Nat Rev Mol Cell Biol. (2011) 12:9–14. doi: 10.1038/nrm3028 21179058 PMC4780047

[88] Zhou R Yazdi AS Menu P Tschopp J . A role for mitochondria in NLRP3 inflammasome activation. Nature. (2011) 469:221–5. doi: 10.1038/nature09663 21124315

[89] Zhong Z Umemura A Sanchez-Lopez E Liang S Shalapour S Wong J . NF-κB restricts inflammasome activation via elimination of damaged mitochondria. Cell. (2016) 164:896–910. doi: 10.1016/j.cell.2015.12.057 26919428 PMC4769378

[90] Katti P Prasad P Masenga SK Venkhatesh P Vue Z Marshall AG . The MICOS complex regulates mitochondrial structure and oxidative stress during age-dependent structural deficits in the kidney. Aging Cell. (2026) 25:e70534. doi: 10.1101/2024.06.09.598108 42115834 PMC13160932

[91] Mammucari C Raffaello A Vecellio Reane D Gherardi G De Mario A Rizzuto R . Mitochondrial calcium uptake in organ physiology: from molecular mechanism to animal models. Pflugers Arch. (2018) 470:1165–79. doi: 10.1007/s00424-018-2123-2 29541860 PMC6060757

[92] Murakami T Ockinger J Yu J Byles V McColl A Hofer AM . Critical role for calcium mobilization in activation of the NLRP3 inflammasome. Proc Natl Acad Sci USA. (2012) 109:11282–7. doi: 10.1073/pnas.1117765109 22733741 PMC3396518

[93] Locati M Curtale G Mantovani A . Diversity, mechanisms, and significance of macrophage plasticity. Annu Rev Pathol. (2020) 15:123–47. doi: 10.1146/annurev-pathmechdis-012418-012718 31530089 PMC7176483

[94] Wang J Gao H Xie Y Wang P Li Y Zhao J . Lycium barbarum polysaccharide alleviates dextran sodium sulfate-induced inflammatory bowel disease by regulating M1/M2 macrophage polarization via the STAT1 and STAT6 pathways. Front Pharmacol. (2023) 14:1044576. doi: 10.3389/fphar.2023.1044576 37144216 PMC10151498

[95] Li T Li Q Liu S Cao J Mei J Gong J . Targeted V-type peptide-decorated nanoparticles prevent colitis by inhibiting endosomal TLR signaling and modulating intestinal macrophage polarization. Biomaterials. (2025) 314:122843. doi: 10.1016/j.biomaterials.2024.122843 39321686

[96] Zhang X Yuan Z Wu J He Y Lu G Zhang D . An orally-administered nanotherapeutics with carbon monoxide supplying for inflammatory bowel disease therapy by scavenging oxidative stress and restoring gut immune homeostasis. ACS Nano. (2023) 17:21116–33. doi: 10.1021/acsnano.3c04819 37843108

[97] Wan L Liu J Huang C Wang K Zhu Z Li F . A novel pharmaceutical preparation of Tripterygium wilfordii Hook. f. regulates macrophage polarization to alleviate inflammation in rheumatoid arthritis. J Pharm Pharmacol. (2023) 75:1442–57. doi: 10.1093/jpp/rgad078 37738207

[98] Lawrence T Natoli G . Transcriptional regulation of macrophage polarization: enabling diversity with identity. Nat Rev Immunol. (2011) 11:750–61. doi: 10.1038/nri3088 22025054

[99] Zhang Y Yang W Kumagai Y Loza M Zhang W Park SJ . Multi-omics computational analysis unveils the involvement of AP-1 and CTCF in hysteresis of chromatin states during macrophage polarization. Front Immunol. (2023) 14:1304778. doi: 10.3389/fimmu.2023.1304778 38173717 PMC10761412

[100] Chang ZY Liu HM Leu YL Hsu CH Lee TY . Modulation of gut microbiota combined with upregulation of intestinal tight junction explains anti-inflammatory effect of corylin on colitis-associated cancer in mice. Int J Mol Sci. (2022) 23(5):2667. doi: 10.3390/ijms23052667 35269806 PMC8910903

[101] Hu X Han C Jin J Qin K Zhang H Li T . Integrin CD11b attenuates colitis by strengthening Src-Akt pathway to polarize anti-inflammatory IL-10 expression. Sci Rep. (2016) 6:26252. doi: 10.1038/srep26252 27188220 PMC4870583

[102] Lin Y Yang X Yue W Xu X Li B Zou L . Chemerin aggravates DSS-induced colitis by suppressing M2 macrophage polarization. Cell Mol Immunol. (2014) 11:355–66. doi: 10.1038/cmi.2014.15 24727542 PMC4085517

[103] Huang C Wang J Liu H Huang R Yan X Song M . Ketone body β-hydroxybutyrate ameliorates colitis by promoting M2 macrophage polarization through the STAT6-dependent signaling pathway. BMC Med. (2022) 20:148. doi: 10.1186/s12916-022-02352-x 35422042 PMC9011974

[104] Dong L Shen Y Li H Zhang R Yu S Wu Q . Shared genes of PPARG and NOS2 in Alzheimer's disease and ulcerative colitis drive macrophages and microglia polarization: Evidence from bioinformatics analysis and following validation. Int J Mol Sci. (2023) 24(6):5651. doi: 10.3390/ijms24065651 36982725 PMC10058634

[105] Hu Q Lyon CJ Fletcher JK Tang W Wan M Hu TY . Extracellular vesicle activities regulating macrophage- and tissue-mediated injury and repair responses. Acta Pharm Sin B. (2021) 11:1493–512. doi: 10.1016/j.apsb.2020.12.014 34221864 PMC8245807

[106] Kanno T Katano T Shimura T Tanaka M Nishie H Fukusada S . Krüppel-like factor-4-mediated macrophage polarization and phenotypic transitions drive intestinal fibrosis in THP-1 monocyte models *in vitro*. Med (Kaunas). (2024) 60(5):713. doi: 10.3390/medicina60050713 38792896 PMC11122781

[107] Zhao H Zhou Y Xu J Zhang Y Wang H Zhao C . Short-chain fatty acid-producing bacterial strains attenuate experimental ulcerative colitis by promoting M2 macrophage polarization via JAK/STAT3/FOXO3 axis inactivation. J Transl Med. (2024) 22:369. doi: 10.1186/s12967-024-05122-w 38637862 PMC11025230

[108] Akira S Takeda K . Toll-like receptor signalling. Nat Rev Immunol. (2004) 4:499–511. doi: 10.1038/nri1391 15229469

[109] Lotze MT Tracey KJ . High-mobility group box 1 protein (HMGB1): nuclear weapon in the immune arsenal. Nat Rev Immunol. (2005) 5:331–42. doi: 10.1038/nri1594 15803152

[110] McInnes IB Schett G . The pathogenesis of rheumatoid arthritis. N Engl J Med. (2011) 365:2205–19. doi: 10.1056/nejmra1004965 22150039

[111] Moore KJ Tabas I . Macrophages in the pathogenesis of atherosclerosis. Cell. (2011) 145:341–55. doi: 10.1016/j.cell.2011.04.005 21529710 PMC3111065

[112] Seyed Tabib NS Madgwick M Sudhakar P Verstockt B Korcsmaros T Vermeire S . Big data in IBD: big progress for clinical practice. Gut. (2020) 69:1520–32. doi: 10.1136/gutjnl-2019-320065 32111636 PMC7398484

[113] Roelands J van der Ploeg M Ijsselsteijn ME Dang H Boonstra JJ Hardwick JCH . Transcriptomic and immunophenotypic profiling reveals molecular and immunological hallmarks of colorectal cancer tumourigenesis. Gut. (2023) 72:1326–39. doi: 10.1136/gutjnl-2022-327608 36442992 PMC10314051

[114] Blum SM Zlotoff DA Smith NP Kernin IJ Ramesh S Zubiri L . Immune responses in checkpoint myocarditis across heart, blood and tumour. Nature. (2024) 636:215–23. doi: 10.1038/s41586-024-08105-5 39506125 PMC12952943

[115] Chathuranga K Rathnapala P Weerawardhana A Kim TH Seong Y Chathuranga WAG . The E3 ubiquitin ligase MARCH2 controls TNF-α mediated inflammation by autoubiquitination. Cell Commun Signal. (2025) 23:257. doi: 10.1186/s12964-025-02260-6 40450320 PMC12126869

[116] Peters LA Perrigoue J Mortha A Iuga A Song WM Neiman EM . A functional genomics predictive network model identifies regulators of inflammatory bowel disease. Nat Genet. (2017) 49:1437–49. doi: 10.1038/ng.3947 28892060 PMC5660607

[117] Huang J Li L Xu L Feng L Wang Y Sik AG . Methyl 3-bromo-4,5-dihydroxybenzoate attenuates inflammatory bowel disease by regulating TLR/NF-κB pathways. Mar Drugs. (2025) 23(1):47. doi: 10.3843/glowm.412693 PMC11766471 39852549

[118] Liu J Wang F Luo H Liu A Li K Li C . Protective effect of butyrate against ethanol-induced gastric ulcers in mice by promoting the anti-inflammatory, anti-oxidant and mucosal defense mechanisms. Int Immunopharmacol. (2016) 30:179–87. doi: 10.1016/j.intimp.2015.11.018 26604089

[119] Yu JS Huang T Zhang Y Mao XT Huang LJ Li YN . Substrate-specific recognition of IKKs mediated by USP16 facilitates autoimmune inflammation. Sci Adv. (2021) 7(3):eabc4009. doi: 10.1126/sciadv.abc4009 33523871 PMC7806237

[120] Jha AK Huang SC Sergushichev A Lampropoulou V Ivanova Y Loginicheva E . Network integration of parallel metabolic and transcriptional data reveals metabolic modules that regulate macrophage polarization. Immunity. (2015) 42:419–30. doi: 10.1016/j.immuni.2015.02.005 25786174

[121] Huang B Chen Z Geng L Wang J Liang H Cao Y . Mucosal profiling of pediatric-onset colitis and IBD reveals common pathogenics and therapeutic pathways. Cell. (2019) 179:1160–1176.e24. doi: 10.1016/j.cell.2019.10.027 31730855

[122] Lu H Zhang C Wu W Chen H Lin R Sun R . MCPIP1 restrains mucosal inflammation by orchestrating the intestinal monocyte to macrophage maturation via an ATF3-AP1S2 axis. Gut. (2023) 72:882–95. doi: 10.1136/gutjnl-2022-327183 37015751

[123] Nitzan M Brenner MP . Revealing lineage-related signals in single-cell gene expression using random matrix theory. Proc Natl Acad Sci USA. (2021) 118(11):e1913931118. doi: 10.1073/pnas.1913931118 33836557 PMC7980374

[124] Rutgeerts P Sandborn WJ Feagan BG Reinisch W Olson A Johanns J . Infliximab for induction and maintenance therapy for ulcerative colitis. N Engl J Med. (2005) 353:2462–76. doi: 10.1056/nejmoa050516 16339095

[125] Sandborn WJ Su C Panes J . Tofacitinib as induction and maintenance therapy for ulcerative colitis. N Engl J Med. (2017) 377:496–7. doi: 10.1056/nejmoa1606910 28767341

[126] Feagan BG Sandborn WJ Gasink C Jacobstein D Lang Y Friedman JR . Ustekinumab as induction and maintenance therapy for Crohn's disease. N Engl J Med. (2016) 375:1946–60. doi: 10.1056/nejmoa1602773 27959607

[127] Madan U Verma B Awasthi A . Cenicriviroc, a CCR2/CCR5 antagonist, promotes the generation of type 1 regulatory T cells. Eur J Immunol. (2024) 54:e2350847. doi: 10.1002/eji.202350847 38643381

[128] Odegaard JI Ricardo-Gonzalez RR Goforth MH Morel CR Subramanian V Mukundan L . Macrophage-specific PPARgamma controls alternative activation and improves insulin resistance. Nature. (2007) 447:1116–20. doi: 10.1038/nature05894 17515919 PMC2587297

[129] Zhang Y Li X Zhang Q Li J Ju J Du N . Berberine hydrochloride prevents postsurgery intestinal adhesion and inflammation in rats. J Pharmacol Exp Ther. (2014) 349:417–26. doi: 10.1124/jpet.114.212795 24676878

[130] Chen Z Jiang P Su D Zhao Y Zhang M . Therapeutic inhibition of the JAK-STAT pathway in the treatment of inflammatory bowel disease. Cytokine Growth Factor Rev. (2024) 79:1–15. doi: 10.1016/j.cytogfr.2024.07.008 39179485

[131] Panés J Sandborn WJ Schreiber S Sands BE Vermeire S D'Haens G . Tofacitinib for induction and maintenance therapy of Crohn's disease: results of two phase IIb randomised placebo-controlled trials. Gut. (2017) 66:1049–59. doi: 10.1136/gutjnl-2016-312735 PMC5532457 28209624

[132] Tracey D Klareskog L Sasso EH Salfeld JG Tak PP . Tumor necrosis factor antagonist mechanisms of action: a comprehensive review. Pharmacol Ther. (2008) 117:244–79. doi: 10.1016/j.pharmthera.2007.10.001 18155297

[133] Dinarello CA . Interleukin-1 in the pathogenesis and treatment of inflammatory diseases. Blood. (2011) 117:3720–32. doi: 10.1182/blood-2010-07-273417 21304099 PMC3083294

[134] Alam S Liu Q Liu S Liu Y Zhang Y Yang X . Up-regulated cathepsin C induces macrophage M1 polarization through FAK-triggered p38 MAPK/NF-κB pathway. Exp Cell Res. (2019) 382:111472. doi: 10.1016/j.yexcr.2019.06.017 31229505

[135] Bauché D Joyce-Shaikh B Jain R Grein J Ku KS Blumenschein WM . LAG3(+) regulatory T cells restrain interleukin-23-producing CX3CR1(+) gut-resident macrophages during group 3 innate lymphoid cell-driven colitis. Immunity. (2018) 49:342–352.e5. doi: 10.1016/j.immuni.2018.07.007 30097293

[136] Bolton C Smillie CS Pandey S Elmentaite R Wei G Argmann C . An integrated taxonomy for monogenic inflammatory bowel disease. Gastroenterology. (2022) 162:859–76. doi: 10.1053/j.gastro.2021.11.014 34780721 PMC7616885

[137] Liu C Liu N Zhang T Tu Y . Adoptive immune cell therapy for colorectal cancer. Front Immunol. (2025) 16:1557906. doi: 10.3389/fimmu.2025.1557906 40236691 PMC11996668

[138] Jin R Neufeld L McGaha TL . Linking macrophage metabolism to function in the tumor microenvironment. Nat Cancer. (2025) 6:239–52. doi: 10.1038/s43018-025-00909-2 39962208

[139] Zhou Y Yang M Yan X Zhang L Lu N Ma Y . Oral nanotherapeutics of andrographolide/carbon monoxide donor for synergistically anti-inflammatory and pro-resolving treatment of ulcerative colitis. ACS Appl Mater Interfaces. (2023) 15:36061–75. doi: 10.1021/acsami.3c09342 37463480

[140] Shakespear MR Halili MA Irvine KM Fairlie DP Sweet MJ . Histone deacetylases as regulators of inflammation and immunity. Trends Immunol. (2011) 32:335–43. doi: 10.1016/j.it.2011.04.001 21570914

[141] Guerriero JL Sotayo A Ponichtera HE Castrillon JA Pourzia AL SChad S . Class IIa HDAC inhibition reduces breast tumours and metastases through anti-tumour macrophages. Nature. (2017) 543:428–32. doi: 10.1038/nature21409 28273064 PMC8170529

[142] Nicodeme E Jeffrey KL Schaefer U Beinke S Dewell S Chung CW . Suppression of inflammation by a synthetic histone mimic. Nature. (2010) 468:1119–23. doi: 10.1038/nature09589 21068722 PMC5415086

[143] Belkina AC Denis GV . BET domain co-regulators in obesity, inflammation and cancer. Nat Rev Cancer. (2012) 12:465–77. doi: 10.1038/nrc3256 22722403 PMC3934568

[144] Cannarile MA Weisser M Jacob W Jegg AM Ries CH Rüttinger D . Colony-stimulating factor 1 receptor (CSF1R) inhibitors in cancer therapy. J Immunother Cancer. (2017) 5:53. doi: 10.1186/s40425-017-0257-y 28716061 PMC5514481

[145] Li L Xu T Qi X . Balanced regulation of ROS production and inflammasome activation in preventing early development of colorectal cancer. Immunol Rev. (2025) 329:e13417. doi: 10.1111/imr.13417 39523732

[146] Han X Ding S Jiang H Liu G . Roles of macrophages in the development and treatment of gut inflammation. Front Cell Dev Biol. (2021) 9:625423. doi: 10.3389/fcell.2021.625423 33738283 PMC7960654

[147] Fridman WH Meylan M Pupier G Calvez A Hernandez I Sautès-Fridman C . Tertiary lymphoid structures and B cells: An intratumoral immunity cycle. Immunity. (2023) 56:2254–69. doi: 10.1016/j.immuni.2023.08.009 37699391

[148] Zhong Y Zhang X Chong W . Interleukin-24 immunobiology and its roles in inflammatory diseases. Int J Mol Sci. (2022) 23(2):627. doi: 10.3390/ijms23020627 35054813 PMC8776082

[149] Xiao Q Li X Li Y Wu Z Xu C Chen Z . Biological drug and drug delivery-mediated immunotherapy. Acta Pharm Sin B. (2021) 11:941–60. doi: 10.1016/j.apsb.2020.12.018 33996408 PMC8105778

[150] Au KM Wilson JE Ting JP Wang AZ . An injectable subcutaneous colon-specific immune niche for the treatment of ulcerative colitis. Nat BioMed Eng. (2024) 8:1243–65. doi: 10.1038/s41551-023-01136-9 38049469

[151] Zhang F Xia Y Su J Quan F Zhou H Li Q . Neutrophil diversity and function in health and disease. Signal Transduct Target Ther. (2024) 9:343. doi: 10.1038/s41392-024-02049-y 39638788 PMC11627463

[152] Chen X Bao S Liu M Han Z Tan J Zhu Q . Inhibition of HMGB1 improves experimental mice colitis by mediating NETs and macrophage polarization. Cytokine. (2024) 176:156537. doi: 10.1016/j.cyto.2024.156537 38325140

[153] Lu H Suo Z Lin J Cong Y Liu Z . Monocyte-macrophages modulate intestinal homeostasis in inflammatory bowel disease. biomark Res. (2024) 12:76. doi: 10.1186/s40364-024-00612-x 39095853 PMC11295551

[154] Xiao L Zhang L Guo C Xin Q Gu X Jiang C . Find me" and "Eat me" signals: tools to drive phagocytic processes for modulating antitumor immunity. Cancer Commun (Lond). (2024) 44:791–832. doi: 10.1002/cac2.12579 38923737 PMC11260773

[155] Ramos C Oehler R . Clearance of apoptotic cells by neutrophils in inflammation and cancer. Cell Death Discov. (2024) 10:26. doi: 10.1038/s41420-024-01809-7 38218739 PMC10787834

[156] Xie M Yuan K Zhang Y Zhang Y Zhang R Gao J . Tumor-resident probiotic Clostridium butyricum improves aPD-1 efficacy in colorectal cancer models by inhibiting IL-6-mediated immunosuppression. Cancer Cell. (2025) 43:1885–1901.e10. doi: 10.1016/j.ccell.2025.07.012 40780216

[157] Ma D Zhang Y Zhang J Shi J Gao S Long F . Outer membrane vesicles derived from probiotic Escherichia coli Nissle 1917 promote metabolic remodeling and M1 polarization of RAW264.7 macrophages. Front Immunol. (2025) 16:1501174. doi: 10.3389/fimmu.2025.1501174 40510339 PMC12159019

[158] Gronke K Hernández PP Zimmermann J Klose CSN Kofoed-Branzk M Guendel F . Interleukin-22 protects intestinal stem cells against genotoxic stress. Nature. (2019) 566:249–53. doi: 10.1038/s41586-019-0899-7 30700914 PMC6420091

[159] Peng X Hou P Chen Y Dai Y Ji Y Shen Y . Preclinical evaluation of 3D185, a novel potent inhibitor of FGFR1/2/3 and CSF-1R, in FGFR-dependent and macrophage-dominant cancer models. J Exp Clin Cancer Res. (2019) 38:372. doi: 10.1186/s13046-019-1357-y 31438996 PMC6704710

[160] Schlechter BL Stebbing J . CCR5 and CCL5 in metastatic colorectal cancer. J Immunother Cancer. (2024) 12(5):e008722. doi: 10.1136/jitc-2023-008722 38719543 PMC11086517

[161] Popēna I Ābols A Saulīte L Pleiko K Zandberga E Jēkabsons K . Effect of colorectal cancer-derived extracellular vesicles on the immunophenotype and cytokine secretion profile of monocytes and macrophages. Cell Commun Signal. (2018) 16:17. doi: 10.1186/s12964-018-0229-y PMC5937830 29690889

[162] Baumgart DC Sandborn WJ . Crohn's disease. Lancet. (2012) 380:1590–605. doi: 10.3238/arztebl.2009.0123 22914295

[163] Rieder F Fiocchi C Rogler G . Mechanisms, management, and treatment of fibrosis in patients with inflammatory bowel diseases. Gastroenterology. (2017) 152:340–350.e6. doi: 10.1053/j.gastro.2016.09.047 27720839 PMC5209279

[164] Hanauer SB Feagan BG Lichtenstein GR Mayer LF Schreiber S Colombel JF . Maintenance infliximab for Crohn's disease: the ACCENT I randomised trial. Lancet. (2002) 359:1541–9. doi: 10.1016/s0140-6736(02)08512-4 12047962

